# Eusociality Shapes Convergent Patterns of Molecular Evolution across Mitochondrial Genomes of Snapping Shrimps

**DOI:** 10.1093/molbev/msaa297

**Published:** 2020-11-19

**Authors:** Solomon T C Chak, Juan Antonio Baeza, Phillip Barden

**Affiliations:** 1 Department of Biological Sciences, New Jersey Institute of Technology, Newark, NJ; 2 Department of Biological Sciences, SUNY College at Old Westbury, Old Westbury, NY; 3 Department of Biological Sciences, Clemson University, Clemson, SC; 4 Smithsonian Institution, Smithsonian Marine Station at Fort Pierce, Fort Pierce, FL; 5 Departamento de Biología Marina, Facultad de Ciencias del Mar, Universidad Católica del Norte, Coquimbo, Chile; 6 Division of Invertebrate Zoology, American Museum of Natural History, New York, NY

**Keywords:** crustacean, decapod, eusociality, genome evolution, low-coverage whole-genome sequencing, shrimp

## Abstract

Eusociality is a highly conspicuous and ecologically impactful behavioral syndrome that has evolved independently across multiple animal lineages. So far, comparative genomic analyses of advanced sociality have been mostly limited to insects. Here, we study the only clade of animals known to exhibit eusociality in the marine realm—lineages of socially diverse snapping shrimps in the genus *Synalpheus*. To investigate the molecular impact of sociality, we assembled the mitochondrial genomes of eight *Synalpheus* species that represent three independent origins of eusociality and analyzed patterns of molecular evolution in protein-coding genes. Synonymous substitution rates are lower and potential signals of relaxed purifying selection are higher in eusocial relative to noneusocial taxa. Our results suggest that mitochondrial genome evolution was shaped by eusociality-linked traits—extended generation times and reduced effective population sizes that are hallmarks of advanced animal societies. This is the first direct evidence of eusociality impacting genome evolution in marine taxa. Our results also strongly support the idea that eusociality can shape genome evolution through profound changes in life history and demography.

## Introduction

Eusociality is a conspicuous and ecologically impactful behavioral syndrome (Wilson 2012) that has convergently arisen at least 17 times in arthropods (Crozier and Pamilo 1996). Eusocial lineages are thought to represent the apex of animal societal organization and a reproductive division of labor is central to the transition to colony life. Within a eusocial colony, a single or subset of individuals perform all reproductive duties whereas others forgo their own reproduction and undertake tasks related to brood care, nest maintenance, and the acquisition of resources. This social organization can produce dramatic changes in phenotype including the evolution of specialized subcastes, extended lifespans, and chemosensory elaboration (Wilson and Hölldobler 2009). Concomitant with phenotypic evolution, genomic analyses indicate that there are unique signatures of molecular evolution in eusocial lineages relative to solitary relatives (Rehan and Toth 2015).

Convergent phenotypes, including those related to sociality, have been shown to produce concurrent changes in nuclear (Parker et al. 2013; Berens et al. 2015; Sackton et al. 2019; He et al. 2020) and mitochondrial genomes (e.g., Tomasco and Lessa 2011; Babbucci et al. 2014). With the advent of low-cost sequencing, comparative genomic analyses have revealed genomic patterns that appear to be linked to advanced eusociality, such as expansions in gene families and elevated substitution rates in nuclear genes (Kapheim et al. 2015; Harrison et al. 2018). A comparison across whole genomes of bees revealed that nuclear genes showing accelerated molecular evolution in species with increased social complexity are under relaxed purifying (i.e., elevated ratio of nonsynonymous to synonymous substitution) and directional selection (Kapheim et al. 2015). Similarly, a recent analysis of 3,236 ortholog genes across 169 Hymenoptera species found signs of relaxed purifying selection in eusocial lineages in more than 50% of these genes (Weyna and Romiguier 2020). Beyond nuclear genes, Bromham and Leys (2005) compared a small number of mitochondrial protein-coding genes (PCGs) across 25 pairs of eusocial and noneusocial species of ants, bees, and termites, as well as shrimps and mole rats. These authors did not find a universal effect of sociality on substitution rates in their data set, but found elevated rates between pairs of a highly eusocial species and a distantly related noneusocial species. A lack of association between sociality and substitution rate in mitochondrial genes in this study may reflect the diversity among lineages used in the analysis—the pairs of sampled species have distinct ecologies beyond social behavior (e.g., including parasitic lifestyles) and so other factors may be affecting substitution rate. Therefore, although molecular analyses of convergence may uncover consistent trends in genome evolution (Christin et al. 2010), comparisons of distant relatives may be subject to noise due to highly dissimilar ecologies and phylogenetic histories. A distinct but complimentary approach is to test the effect of sociality on molecular rates of evolution in a single clade with multiple recent and independent eusocial origins.

The sponge-dwelling snapping shrimps in the genus *Synalpheus* are the only known group of marine animals to have evolved advanced sociality (Duffy 1996; Hultgren et al. 2017) and have at least four independent origins of eusociality (Chak et al. 2017). The nine described eusocial species are found in the West Atlantic “*Synalpheus gambarelloides*” group, a relatively young lineage that radiated relatively recently, between ∼5 and 7 Ma (Morrison et al. 2004). The multiple recent origins of eusociality in shrimps contrast with those of social insects such as ants and termites, each with single ancient origins that took place ∼120–∼150 Ma (Moreau and Bell 2013; Barden and Grimaldi 2016; Engel et al. 2016; Evangelista et al. 2019). Bees and wasps include multiple origins of sociality, but sister group relationships have proven difficult to resolve in some cases and sister lineages exhibit dissimilar ecologies that may themselves impact molecular evolution (Gibbs et al. 2012; Peters et al. 2017). *Synalpheus* shrimps, therefore, represent a unique lens to examine the genomic causes and consequences of complex social behavior among closely related congeners that exhibit multiple origins of eusociality.

Here, we assessed whether there are convergent patterns of selection and changes in substitution rates in mitochondrial PCGs across recent, independent origins of eusociality in *Synalpheus* shrimps. Some eusocial lineages are known to exhibit distinct molecular evolution related to life history (Bromham and Leys 2005; Romiguier et al. 2014; Weyna and Romiguier 2020). Here specifically, we predict that lower effective population size, longer generation times, and shifts in metabolic activity shape rates and patterns of molecular evolution in social shrimps. We examined the changes in nonsynonymous and synonymous substitution rates (d*N* and d*S* hereafter) among eusocial and noneusocial species in 13 mitochondrial PCGs. Although it may be difficult to assess whether molecular evolution in nuclear genes represents causes or consequences of eusociality (Kapheim et al. 2015; Rubenstein et al. 2019), changes in mitochondrial PCGs may be more simple to interpret. Because mitochondrial PCGs are coding for conserved cell respiratory functions (Boore 1999), changes in these genes are unlikely to underpin the evolution of eusociality and, instead, may reflect the indirect genomic changes that are driven by demographic, life history, or physiological shifts that accompany the evolution of eusociality (Bromham and Leys 2005; Romiguier et al. 2014; Weyna and Romiguier 2020). We also examined the direction and magnitude of natural selection using the d*N*/d*S* ratio (denoted by ω hereafter; also referred to as *K*_A_/*K*_S_), where values equal to 1, <1, or >1 indicates the presence of no selection, purifying (negative) selection, or diversifying (positive) selection, respectively. Further, we tested for signs of positive selection and relaxed purifying selection at evolutionary branches leading to eusociality. We explain the observed changes we recover as they relate to putative life history changes in eusocial species. Our results provide the first direct evidence of eusociality shaping genome evolution in marine taxa.

## Results

### Mitogenome Annotation and Phylogenetic Analysis

We analyzed eight mitogenomes from four eusocial species (*S. chacei, S. filidigitus, S. microneptunus*, and *S. regalis*) and four noneusocial species (*S. carpenteri, S. hoetjesi, S. kensleyi*, and *S. pandionis*). These included seven newly assembled mitogenomes and a recently published mitogenome of *S. microneptunus* (Chak et al. 2020). All mitogenomes contain 13 PCGs, two ribosomal RNA genes (*rrnS* [12S ribosomal RNA] and *rrnL* [16S ribosomal RNA]), and 22 transfer RNA (tRNA) genes (supplementary table S2, Supplementary Material online). The seven newly assembled mitogenomes had 44–155× coverage and were automatically (in *NOVOPlasty*) or manually circularized in five and two species, respectively. The lengths of these mitogenomes are similar to and synteny is identical to that reported for *S. microneptunus* (Chak et al. 2020).

**Fig. 1.**
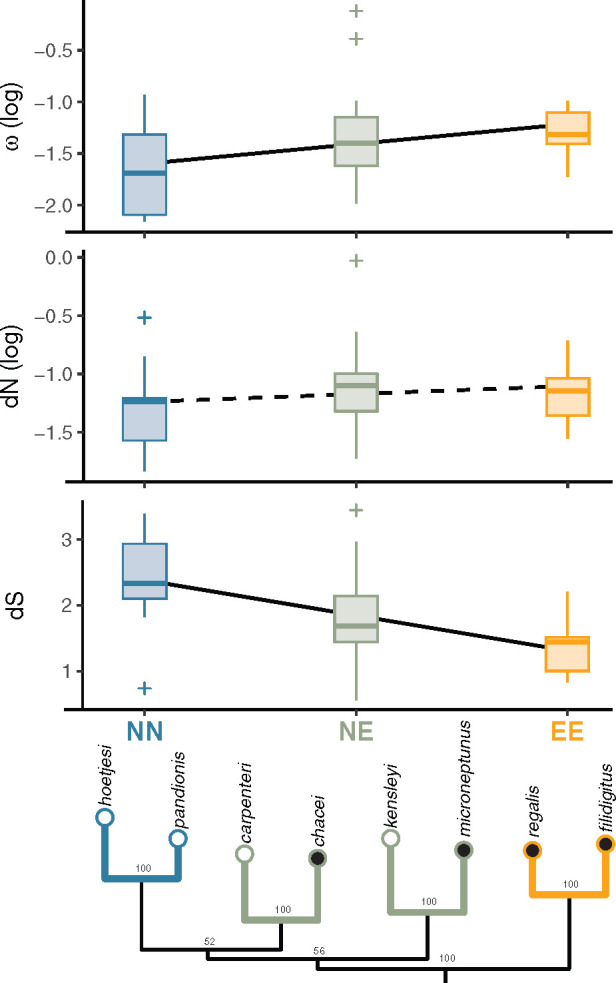
Pairwise ω, d*N*, and d*S* of all 13 mitochondrial PCGs across four sister pairs of *Synalpheus* species of different social groups (NN, NE, EE; where N = noneusocial and E = eusocial). Lines represent results of linear regressions, which controlled for PCG length and nonindependence of loci through nucleotide-based genetic distances. Solid and dashed lines represent significant (*P* < 0.05) and nonsignificant slopes (*P* > 0.05), respectively. The ML phylogenetic tree is shown at the bottom. Colors of terminal branches correspond with social group of the sister pairs. Open and solid dots at the tips indicate noneusocial and eusocial species, respectively.

As a basis of the analyses of substitution rates and selection, we used all 13 mitochondrial PCGs to reconstruct the phylogenetic history of the eight *Synalpheus* mitogenomes. Maximum likelihood (ML) phylogenetic analysis based on nucleotide alignment resulted in a well-resolved phylogenetic tree (fig. 1 and supplementary fig. S1, Supplementary Material online). The general time reversible substitution model with inverse gamma-distributed rate variation across sites was identified as the best model for each PCG (supplementary table S3, Supplementary Material online). Bootstrap supports at the basal nodes of *Synalpheus* were lower, reflecting the inconsistent placement of major *Synalpheus* subclades in our amino acid-based tree (supplementary fig. S1, Supplementary Material online), and the similarly modest basal resolution of the *Synalpheus* clade in earlier phylogenetic studies (Chak et al. 2017). In our downstream analyses, we used both the nucleotide and amino acid-based trees when needed and used the branch lengths only within major *Synalpheus* subclades, which are well resolved and consistently supported, to avoid the uncertainty in basal relationship.

### Comparison of *Synalpheus* Sister Pairs

We analyzed the pairwise values of d*N*, d*S*, and ω within the eight *Synalpheus* species using *KaKs_Calculator* v2.0 (Wang et al. 2010)*.* Because these pairwise values are potentially confounded by the shared phylogenetic history across species (Dunn et al. 2018), we restricted the analysis to only four pairs of sister species, each within a major *Synalpheus* clade (fig. 1). These four major clades were consistently recovered in our mitochondrial trees and in other independent studies using different molecular markers (Morrison et al. 2004; Hultgren and Duffy 2011; Hultgren et al. 2014; Chak et al. 2017). We grouped these pairs into three social groups: one pair of noneusocial species (NN: *S. hoetjesi* vs. *S. pandionis*), two pairs of eusocial and noneusocial species (NE: *S. carpenteri* vs. *S. chacei*; *S. kensleyi* vs. *S. microneptunus*), and one pair of eusocial species (EE: *S. filidigitus* vs. *S. regalis*). Then, we used a general linear model to test whether d*N*, d*S*, and ω increased or decreased along a discrete gradient of social groups (NN–NE–EE).

Across four sister pairs of *Synalpheus*, pairwise values of d*S*, and ω, but not d*N*, were significantly affected by social groups (NN, NE, EE), after controlling for the effect of PCG length and genetic distance between species (fig. 1). Pairwise values of d*N* showed a statistically nonsignificant, increasing trend NN<NE<EE along social groups (*F*_1,48_ = 1.30, *P* = 0.259). In turn, d*S* showed a decreasing and statistically significant trend NN>NE>EE (*F*_1,48_ = 20.98, *P* = 0.00003). Values of ω were less than one in all pairs but showed an increasing and statistically significant trend NN<NE<EE (*F*_1,48_ = 8.90, *P* = 0.005). Further, longer PCGs had higher d*N*, lower d*S*, and higher ω than shorter PCGs (*F*_1,45_ = 211.31, 5.32, and 0.15, and *P* = 0.002, 0.025, and 0.0004, respectively). This indicates that the effect of social groups on d*N*, d*S*, and ω is more prominent in longer PCGs. Analyses between pairs of *Synalpheus* and *Alpheus* species showed similar trends, although lacking statistical significance (see Supplementary Material online). In summary, these results suggest that eusocial *Synalpheus* species tended to have lower synonymous substitution rates (d*S*) and showed signs of relaxed purifying selection (higher values of ω), whereas nonsynonymous substitution rates (d*N*) were higher but statistically nonsignificant in eusocial species.

### Within-Gene Comparison among *Synalpheus* Sister Pairs

**Fig. 2.**
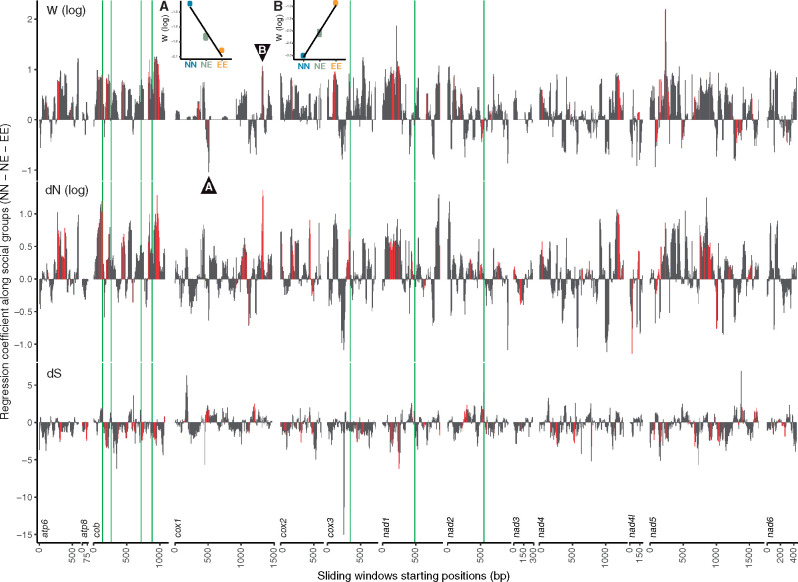
Regression coefficients of ω, d*N*, and d*S* along social groups (NN–NE–EE) at sliding windows along PCGs between four sister pairs of *Synalpheus*. Each bar represents a sliding window of 57 bp long and 6 bp apart. Red bars represent significant regression coefficients (*P* < 0.05) in linear regression models controlling for the effect of genetic distance. A and B insets demonstrate the regression at sliding windows that begin at cox1-499 and cox1-1315, showing positive (NN<NE<EE) and negative (NN>NE>EE) regression coefficients (i.e., predicted slope), respectively. Green lines represent codon positions that showed positive selection in eusocial branches in PAML.

To investigate within-gene selection, we employed a sliding-windows approach to compare d*N*, d*S*, and ω between short sections within each PCG (57 bp long and 6 bp apart) among species pairs. Comparing four sister pairs of *Synalpheus* species, most sliding windows along the 13 PCGs showed the same trend as observed across the whole PCG (fig. 2) (68%, 65%, and 63% of the windows, respectively, for d*N*, d*S*, and ω across the combined length of 13 PCGs). Social group had a significant effect on a small number of windows (3%, 5%, and 6%, respectively). Only 1% of windows showed a significant and opposite trend from that of whole PCG. Within each PCG, we also noted that shorter PCGs including *atp8*, and *nad3*, and to some extent, *nad4* and *nad6*, showed mostly opposite trends in d*N* relative to other PCGs. This agrees with the pairwise analysis of whole PCG which showed that PCG length had a significant effect on d*N* and explains why those analyses failed to detect a significant increase of d*N* in eusocial species across all PCGs. Analyses between pairs of *Synalpheus* and *Alpheus* species showed similar trends (see Supplementary Material online).

### Signs of Selection: PAML

Although the above analyses tested the effect of social groups based on pairs of species, the effect of eusociality on d*N*, d*S*, and ω can also be tested at branches leading to eusocial species because they may have experienced dramatic changes in life history and selective pressures (Bromham and Leys 2005; Romiguier et al. 2014). Therefore, we used the “branch models” in *PAML* (Yang 1997, 2007) to test whether branches leading to eusocial species have different ω values than those leading to noneusocial species.

**Fig. 3.**
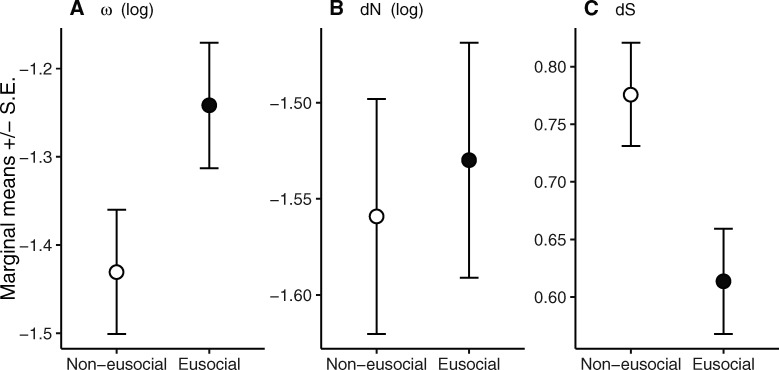
Marginal means of (*A*) ω, (*B*) d*N*, and (*C*) d*S* of terminal branches leading to eusocial and noneusocial species, after controlling for the difference in PCG length. Values were predicted by the free-ratio model of PAML.

Free-ratios models, in which ω values were free to vary in each branch, were better supported than fixed-ratio models, in which ω value was the same in all branches, for most PCGs (except *nad2* and *nad6*) (supplementary table S4, Supplementary Material online). This suggests that ω is not uniform across branches. Compared with terminal branches leading to noneusocial species, those leading to eusocial species had slightly higher but not statistically significant d*N* (*F*_1,90_ = 0.11, *P* = 0.735), significantly lower d*S* (*F*_1,90_ = 6.44, *P* = 0.013), and marginally higher ω (*F*_1,90_ = 3.55, *P* = 0.062), after controlling for PCG length (fig. 3). Within PCGs, most PCGs also exhibited lower d*S* in eusocial terminal branches, except for *atp8* and *cox2* (supplementary table S5, Supplementary Material online). The trends in the free-ratio model are the same to those found when analyzing the *Synalpheus* sister pairs.

The two-ratio models, in which the foreground (eusocial branches) had a different ω value from the background (all other branches), were better supported than the fixed-ratio models for seven PCGs (*atp6, cob, cox1, cox3, nad1, nad4*, and *nad5*) (supplementary table S4, Supplementary Material online). In the two-ratio models, values of ω estimated from the eusocial lineages were always greater than those from the background in all PCGs. This agrees with the observations of elevated ω when analyzing the *Synalpheus* sister pairs and, together, points to the presence of relaxed purifying selection in eusocial species.

Then, we ran a “branch-site test of positive selection” (Yang 2005; Zhang et al. 2005) to test whether a proportion of codons (i.e., sites) in eusocial lineages (foreground) underwent positive selection compared with those in noneusocial lineages (background) for each PCG. The alternative model (“model A”; which estimates the proportions and ω in four classes among sites, allowing eusocial branches to have ω ≥ 1 at some sites) and the null model were equally supported for all PCGs, except for *cox2* in which the null model was marginally better supported. Therefore, there was no signal for positive selection at any PCG. From model A, the majority of the sites had ω < 1 (mean = 89.14%, SD = 7.60%) in both the foreground and background branches across PCGs. A small number of sites had ω = 1 for both the foreground and background (mean = 6.32%, SD = 4.94). Very few sites had ω > 1 in the foreground and ω < 1 or ω = 1 in the background (mean = 4.15%, SD = 3.78%; mean = 0.39%, SD = 0.58%, respectively). Therefore, most sites along the PCGs were under purifying selection, with a few sites showing potential signs of positive selection.

Bayes empirical Bayes (BEB) inference (Yang 2005) identified seven codons that were under positive selection in eusocial lineages when compared against the background branches (with posterior probability > 95%) (fig. 3 and supplementary table S6, Supplementary Material online). Four of these positively selected codons were within *cob*. One codon (codon position 214) was an exposed ω-loop. The other codons (starting at codon positions 47, 214, and 296) were buried α-helixes. The three α-helix codons were within sliding windows which showed significantly higher ω in eusocial lineages in *Synalpheus* sister pairs comparison. Between sister pairs of eusocial and noneusocial species, we observed no signs of convergent change in amino acids at these codons. All changes were identified as neutral by *SNAP2*. However, six out of 14 amino acid changes between sister pairs of eusocial and noneusocial species were radical replacement (i.e., those between different classes of amino acids), whereas four had conservative replacements and four has no change. Therefore, the few positively selected codons did not show convergent change in eusocial species, but whether these independent changes affect protein function remains to be addressed using experimental work.

### Signs of Selection: *HyPhy*

We used several methods available in *HyPhy* (Kosakovsky Pond et al. 2020) to test, among the eight *Synalpheus* species, whether branches leading to eusocial species show signs of positive selection and relaxed purifying selection. The use of *HyPhy* methods may be especially suited to our aim because these methods allow d*S* to vary across sites and/or branches instead of constraining it to 1 as in *PAML* (Pond and Muse 2005). Therefore, these methods may more accurately model the effect of life history changes that affect d*S* in each studied clade.

**Fig. 4.**
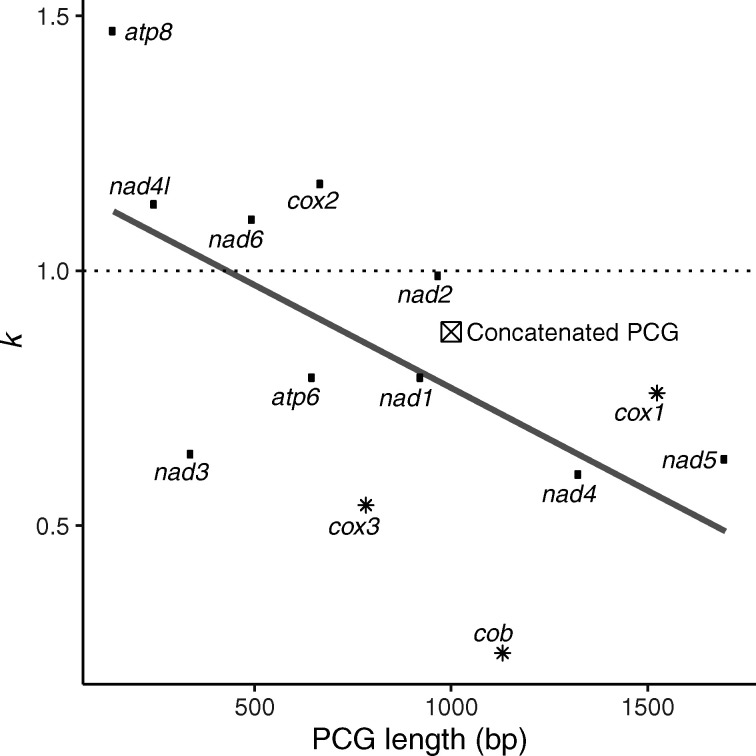
Selection intensity parameter (*k*) across PCGs of different lengths. *k* > 1 indicates an increased selection strength, whereas *k* < 1 indicates a relaxed selection strength at eusocial branches. Gray line represents linear regression line (*P* = 0.030). Asterisks represent PCGs in which the model with *k* ≠ 1 is better supported. Square with cross indicates the value of *k* for the concatenated PCG in which a model with k ≠ 1 is also better supported.

There were no signs of positive selection in eusocial branches in a proportion of sites (*aBSREL*) (Smith et al. 2015) or at any eusocial branch for at least one site (*BUSTED*) (Murrell et al. 2015). In contrast, we found clear signs of relaxed selection in eusocial lineages based on *RELAX* (fig. 4 and supplementary table S7, Supplementary Material online) (Wertheim et al. 2015). The concatenated PCGs showed relaxed purifying selection in eusocial branches (selection intensity parameter *k *=* *0.88, *P* < 0.015). Within each PCG, three genes (*cob*, *cox1*, and *cox3*) showed strong signals of relaxed purifying selection in eusocial branches (*k *=* *0.25–0.76, where *k *>* *1 indicates an increased selection strength, whereas *k *<* *1 indicates relaxation) in which the alternative model (*k *≠* *1) was better supported than the null model (*k *=* *1) (*P* < 0.001). Six PCGs also showed variable signs of relaxed purifying selection (*k *=* *0.61–0.99) but the alternative and null models had similar likelihoods. Finally, four shorter PCGs showed minor signs of more intense purifying selection (*atp8, cox2, nad4l*, and *nad6*; *k *=* *1.10–1.47), but the alternative models were not better supported than the null model. Further, values of *k* tended to reduce in longer PCGs (ANOVA, *F*_1,11_ = 6.227, *P* = 0.030), meaning that relaxed purifying selection is more prominent in longer PCGs. This trend was also found in the pairwise analysis of *Synalpheus* sister pairs, where longer genes exhibited higher ω. In the three genes where relaxed purifying selection was significant, a majority of the codons showed strong purifying selection (84.69%, 94.92%, and 73.89%, for *cob*, *cox1*, and *cox3*, respectively), whereas the other codons showed relaxed purifying selection (15.31%, 4.65%, and 26.11%, respectively) or neutrality (ω = 0; 0%, 0.43%, and 0%, respectively). We observed a similar trend across all PCGs, an average of 62.07% (SD = 34.22%) of codons showed strong purifying selection, 35.48% (SD = 31.94%) showed relaxed purifying selection, and 2.45% (SD = 3.86%) showed neutrality or positive selection (ω > 1). The signs of relaxed purifying selection here agree with the observations of elevated ω in the analysis of *Synalpheus* sister pairs, free-ratio models and the two-ratio model of *PAML*.

## Discussion

In our analyses of the mitochondrial PCGs of eight *Synalpheus* species, we found convergent changes in substitution rates and selection strength linked to eusocial behavior. Eusocial *Synalpheus* lineages showed a marginal increase in nonsynonymous substitution rate (d*N*) and a clear decrease in the synonymous substitution rate (d*S*). Importantly, eusocial shrimps exhibit higher values of ω (d*N*/d*S* ratio) as shown by multiple independent analyses. These ω values are partly impacted by decreased d*S* we observed, but also may reflect signs of relaxed purifying selection that corroborate with similar trends reported in eusocial insects (Bromham and Leys 2005; Kapheim et al. 2015; Weyna and Romiguier 2020). Although there is an overall background of relaxed selective pressure, we observed seven potentially positively selected sites associated with the evolution of eusocial behavior in four PCGs. The convergent patterns in molecular evolution we report here are more likely the consequences of eusociality rather than drivers of complex social behavior. Mitochondrial PCGs code for the same conserved cell respiratory functions across all eukaryotes regardless of their ecology and social organizations (Lang et al. 1999). In contrast to mitochondrial genes, nuclear genes can code for both conserved functions and novel functions associated with the evolution of eusociality. Agreeing with the emerging new view on the coevolution of genome architecture and sociality (Rubenstein et al. 2019), we argue that these shifts in genome evolution can be explained by changes in demographic history and life history associated with the evolution of eusociality, particularly generation time, effective population size, and metabolic adaptation.

The generation time of eusocial species is generally longer than noneusocial species and can affect substitution rates. In eusocial insects, reproductive queens may live an order of magnitude longer than nonreproductive workers (Keller and Genoud 1997); a similar pattern is also found in social mammals (Schmidt et al. 2013, 2014). Beyond the longevity of the queen, delayed production of first reproductives in newly found colonies (Thorne et al. 2002; Ingram et al. 2013) may also extend generation time in eusocial species. Broadly, eusocial insect colonies do not produce new reproductive individuals, who ultimately dictate generation time, until reaching a given colony size. In eusocial shrimps, very little is known about a queen’s lifespan or a colony’s growth and reproductive output, but an increased generation time is suspected (Hultgren et al. 2017). Across eusocial *S. elizabethae* colonies, where multiple queens per colony is common, the number of queens increases with colony size (Chak et al. 2015), which suggests that a colony’s reproductive output may be lower in smaller, and presumably younger, colonies. According to the generation time hypothesis, species with longer generation times should have a lower nucleotide mutation rate per year due to fewer DNA replication errors per unit time (Laird et al. 1969). Our data in shrimps showed a clear decrease in the synonymous substitution rate (d*S*) in eusocial lineages across all of our analyses. This pattern indicates that eusocial species have a reduced rate of neutral substitution or mutation, which agrees with a predicted increase in generation time.

If longer generation time is the only factor affecting the rate of molecular evolution in eusocial species, d*N* and d*S* should both be reduced, because these rates are based on the nucleotide mutation rate (Stoletzki and Eyre-Walker 2011). However, d*N* remains constant if not slightly increased in eusocial lineages. This suggests that factors other than generation time are affecting molecular evolution in this system. Eusociality is characterized by an intense reproductive division of labor between the queen(s) and nonreproductive workers. Therefore, the effective population size (*N*_e_) is essentially reduced to the small subset of the population (i.e., the queens) that contributes to breeding. Indeed, sociality is shown to be associated with reduced *N*_e_ in Hymenoptera (Romiguier et al. 2014; Weyna and Romiguier 2020) and spiders (Settepani et al. 2017). Similarly, in *Synalpheus* shrimps, demographic inference found lower and more stable *N*_e_ in eusocial species across the four independent origins of eusociality (Chak, Solomon T. C. Harris, Stephen E. Duffy, J. Emmett Hultgren, Kristin M. Rubenstein, Dustin R., submitted). The nearly neutral theory (Ohta 1976; Ohta and Gillespie 1996) predicts that lower *N*_e_ is associated with a higher probability of fixation of slightly deleterious mutations, leading to higher nonsynonymous substitution rates (d*N*). An accumulation of deleterious mutations due to relaxed selection and reduced *N*_e_ has also been observed in domesticated animals (Cruz et al. 2008; Moray et al. 2014). Curiously, although the reduced *N*_e_ in eusocial species would be a plausible explanation for increased d*N*, the longer generation time in eusocial species may be responsible for decreases in d*N*. The combined effect of these two factors could explain the lack of a significant trend in d*N* between eusocial and noneusocial lineages. Additional studies in this and other clades of marine and terrestrial animals that include a range of social organizations are needed to elucidate the relative contributions of each factor to differences in molecular evolution found here.

We suggest that the relatively constant d*N* and a reduction in d*S* in eusocial species, as driven by their longer generation time and lower effective population size, led to an increase in d*N*/d*S* ratio (or ω). Across different independent analyses, we found that eusocial lineages have higher ω which could be indicative of the broad effect of relaxed selection strength in eusocial species across PCGs. Indeed, the program *RELAX*, which tests specifically for underlying relaxed selection (Wertheim et al. 2015), confirmed the presence of significant relaxed selection in the concatenated PCG data set as well as several individual PCGs, and found a general sign of relaxed selection across most genes (fig. 4). A few shorter PCGs tended to have a selection intensity parameter (*k*) slightly above one (although insignificant) and a lower d*N*, indicating a lack of relaxed selection strength at eusocial branches. In general, selective constraints are stronger in shorter genes (Zhang 2000). Therefore, mutations may be more deleterious in shorter mitochondrial genes than in longer and more complex genes, hence these PCGs may be less affected by reduced *N*_e_. Overall, we found support for relaxed purifying selection across most mitochondrial PCGs in eusocial lineages, which can be explained by the reduced effective population sizes and longer generation time in eusocial species.

Metabolic adaptation in eusocial species may lead to positive selection in mitochondrial genes as we have observed seven codons in four PCGs that showed signs of positive selection in eusocial lineages. First, increasing social complexity is generally accompanied by an increased need for communication related to the division of labor, group cohesion, and concerted actions (Leonhardt et al. 2016), which may increase metabolic requirements. Metabolic rate is also correlated with social rank (Røskaft et al. 1986). In eusocial snapping shrimps, workers in a colony respond coordinately to intruders by snapping in concert (Tóth and Duffy 2005); female workers are reproductively suppressed by the presence of the queen but will also compete intensively for the reproductive position when opportunities arise (Chak et al. 2015). Therefore, we do not expect noneusocial snapping shrimp species, which are socially monogamous, to exhibit such an intense “social life” and have an added metabolic requirement. However, higher metabolic rates are expected to increase mutation rate in mtDNA and hence increase d*S* (Baer et al. 2007). Our finding of reduced d*S* suggests that eusocial species may not have higher metabolic rates, at least not as observed from this comparative molecular perspective. Second, metabolic requirements in eusocial species may be related to their life history. In insects, the longer life spans and higher reproductive output in queens suggest that they may have greater oxidative stress resistance (Harman 1956), potentially achieved through changes in mitochondrial proteins involved in cellular respiration. This hypothesis has received mixed empirical support across eusocial species (Parker et al. 2004; Corona et al. 2005; Schmidt et al. 2014). Despite the lack of empirical study on metabolic requirements in *Synalpheus*, the signs of positive selection that we observed in eusocial lineages support a possible link between eusociality and metabolic adaptation.

Although sites identified to be positively selected by BEB inference may represent natural selection acting on eusocial species, they can also be slightly deleterious mutations fixed by drift, pronounced in eusocial species with low *N*_e_. However, our data cannot clearly differentiate between these possibilities. First, the majority of amino acid changes between eusocial and noneusocial sister species are radical changes that result in different physicochemical properties and may have stronger deleterious effects than other codons. The codons with radical amino acid replacements should be removed by purifying selection, and their presence suggests that there may be positive selection on these genes. In particular, four codons in *cob* were identified to be positively selected in eusocial lineages. The gene *cob* codes for the protein cytochrome b, a component of the respiratory chain complex III that is important in the generation of ATP. In humans, mutations in *cob* can result in exercise intolerance (Blakely et al. 2005) and point mutations in *cob* are associated with drug resistance in malaria parasites (Siregar et al. 2008). However, all codons identified under positive selection were predicted to have neutral changes on protein function, although nonsynonymous change could affect protein confirmation via posttranscriptional processing and regulation of RNA (Sauna and Kimchi-Sarfaty 2011). Further, in contrast to *PAML*’s Bayesian inference (BI) of positive selection (Yang 2005), methods in *HyPhy* did not identify any signs of positive selection. The null results from *HyPhy* may be because the approach allows d*S* to vary across sites and/or branches. Indeed, we found that d*S* tended to be lower in terminal branches leading to eusocial species in *PAML*’s free-ratio model. Hence, these positively selected sites identified in *PAML* could be false positives due to the variability of d*S* across branches. Overall, it remains unclear whether these sites are indeed experiencing positive selection at eusocial lineages. A broader sampling of eusocial and noneusocial *Synalpheus* may better clarify the presence of positively selected sites related to eusociality.

On the other hand, the presence of positively selected sites can interfere with selection in linked or neighboring sites (Comeron et al. 2008) due to Hill–Robertson interference (Hill and Robertson 1966), leading to relaxed selection. This effect should be prominent in nonrecombining organelle genomes (McVean and Charlesworth 2000), although there is evidence that mitochondrial genomes do experience occasional recombination in animals (Neiman and Taylor 2009), including crustaceans (Ladoukakis and Zouros 2001). Although PCGs are considered to be strongly linked in the mitogenome, we expect that a positively selected site may interfere most strongly with the neighboring sites resulting in a cluster of sites with relaxed selection around a positively selected site. In our data, although a few codons that were identified to be positively selected in BEB (fig. 2) were within sliding windows that showed relaxed purifying selection (higher ω) in eusocial lineages, such windows were also observed throughout most regions along all PCGs, including PCGs that did not have any positively selected codon. Therefore, the propensity of sliding windows with relaxed purifying selection in eusocial lineages and multiple independent analyses suggest a widespread effect of relaxed purifying selection among mitochondrial PCGs in *Synalpheus*.

Our results join an increasingly large body of evidence that eusociality may convergently shape genome evolution. Known patterns relate to nuclear evolution across distantly related taxa and include expansions and modifications in gene families and gene regulation associated with chemosensation and development. Results here highlight a distinct, but complementary window: mitochondrial consequences of advanced sociality among close relatives during recent origins of eusociality. Moreover, rather than molecular modifications underlying eusociality-linked physiology, our results may be best explained as secondary consequences of advanced social behavior. We identified several convergent patterns of genome evolution among mitochondrial PCGs across three independent origins of eusociality in the shrimp genus *Synalpheus*. Eusocial species showed signs of reduced neutral substitution rate that is consistent with prolonged generation time. We also found possible signs of relaxed selection in eusocial lineages, which are consistent with their smaller effective population sizes. Finally, we found mixed evidence of several sites undergoing positive selection in eusocial lineages that may represent adaptation. Our result provides clear evidence that the evolution of eusociality can, in turn, affect genome evolution through changes in life history and demography.

## Materials and Methods

### Field Collection and Sequencing

We used single specimen samples from four eusocial species (*S. chacei, S. filidigitus, S. microneptunus*, and *S. regalis*) and four noneusocial species (*S. carpenteri, S. hoetjesi, S. kensleyi*, and *S. pandionis*) for DNA extraction and low-coverage whole-genome sequencing. These species included three independent origins of eusociality, as well as two species from each of the four *Synalpheus* major clades (supplementary table S1, Supplementary Material online). The field collection protocol has been reported in Macdonald et al. (2006). We extracted genomic DNA using several walking legs from alcohol-preserved specimens with Qiagen DNeasy Tissue Kits (Qiagen). Extracted DNA was quantified using a Qubit 3.0 Fluorometer with the dsDNA HS assay (ThermoFisher Scientific) and visualized on 2% agarose gels. We provided 1,500 ng of genomic DNA to Novogene (Chula Vista, CA) for TruSeq PCR-free library preparation (Illumina) and 150-bp pair-end sequencing on an Illumina NovaSeq to obtain at least 1× coverage according to published genome size (4.8–11.8 GB) (Jeffery et al. 2016). Low-coverage whole-genome sequencing reads from whole-cell extraction contain a high copy number of extranuclear sequences, and it has been shown to be an efficient and economical approach to assemble complete mitochondrial genomes (Dierckxsens et al. 2016).

### Mitogenome Assembly and Annotation

For the mitogenome assembly of the seven *Synalpheus* species, we used the *NOVOPlasty* pipeline v. 1.2.3 (Dierckxsens et al. 2016) and a “bait” reference mitochondrial genome from *S. microneptunus* (GenBank accession number: MN750781). The newly assembled mitochondrial genomes were first annotated in the MITOS web server (http://mitos.bioinf.uni-leipzig.de) (Bernt et al. 2013) using the invertebrate mitochondrial code. Manual annotation curation, including start and stop codon corrections, was conducted using Expasy (https://web.expasy.org/) (Artimo et al. 2012) and MEGA X (Kumar et al. 2018).

### Phylogenetic Analysis

We used all 13 mitochondrial PCGs to reconstruct the phylogenetic history of the eight *Synalpheus* mitogenomes: seven new assemblies and a published assembly of *S. microneptunus* (Chak et al. 2020). We built ML phylogenetic trees using nucleotide and amino acid alignments of 13 mitochondrial PCGs from eight *Synalpheus* species, five *Alpheus* species (*A. bellulus, A. distinguendus, A. lobidens A. inopinatus*, and *A. randalli*; GenBank accession numbers: MH796167, NC_014883, KP276147, MG551491, and MH796168, respectively), and six outgroup species. The outgroup included two species from each of Pandalidae (*Heterocarpus ensifer*: MG674228 and *Chlorotocus crassicornis*: KY944589), Atyidae (*Neocaridina denticulate*: NC_023823 and *Typhlatya mitchelli*: KX844712), and Palaemonidae (*Macrobrachium rosenbergii*: KY865098 and *Palaemon sinensis*: NC_045090). For nucleotide-based analyses, we aligned each of the 13 PCGs using *Muscle* under default parameters (Edgar 2004). Then, we used *AMAS* (https://github.com/marekborowiec/AMAS) to concatenate the alignment files and produce a partition file (Borowiec 2016). We used *ModelTest-NG* (Darriba et al. 2020) to identify appropriate models for each partition used in ML tree searches. We used *RAxML-NG* (Kozlov et al. 2019) for ML reconstructions with 1,000 bootstrap pseudoreplicates. For amino acid-based analyses, we translated the nucleotide sequences according to the invertebrate mitochondrial codon table using *EMBOSS Transeq* (Madeira et al. 2019), aligned the sequences using *Clustal Omega* (Sievers et al. 2011), and reconstructed ML tree as described above. Trees were visualized in *R* v3.6.0 using the packages *ggtree* (Yu et al. 2017) and *treeio* (Wang et al. 2020). We also reconstructed phylogenies under BI using *MrBayes* (Huelsenbeck and Ronquist 2001), and the resulting trees were identical to the ML trees (see Supplementary Material online).

### Comparison of *Synalpheus* Sister Pairs

We used a ML method implemented in *KaKs_Calculator* v2.0 (Wang et al. 2010) to estimate values of d*N*, d*S*, and ω in 13 mitochondrial PCGs between sister pairs of *Synalpheus* species. We performed the calculations for each PCG separately. And as a consensus metric, we also performed the calculations with a concatenated alignment of all 13 PCGs, which assumes that the PCGs evolved as a single unit. *KaKs*_*Calculator* uses model-selection and model-averaging to compute d*N*, d*S*, and ω values across a group of existing nucleotide substitution models, incorporating variable mutation rates across sites (Zhang et al. 2006; Wang et al. 2010). Therefore, it accounts for heterogeneity in the assumptions made by different substitution models and the differences in estimated substitution rates across substitution models in a likelihood framework.

We analyzed the pairwise values of d*N*, d*S*, and ω within the eight *Synalpheus* species*.* We analyzed only four pairs of sister species, each within a major *Synalpheus* clade. These four major clades were consistently recovered in our mitochondrial trees and in other independent studies using different molecular markers (Morrison et al. 2004; Hultgren and Duffy 2011; Hultgren et al. 2014; Chak et al. 2017). In our mitochondrial trees, the relationships among these four clades were not consistent between amino acid and nucleotide data sets (see results), potentially because amino acid data may have lower phylogenetic informativeness (smaller number of characters than nucleotide data) and thus, the use of amino acids is likely to diminish phylogenetic resolution (Simmons et al. 2002). Despite that, our method of comparing only sister species within a clade avoided relying on a single topology. We grouped these pairs into three social groups: one pair of noneusocial species (NN: *S. hoetjesi* vs. *S. pandionis*), two pairs of eusocial and noneusocial species (NE: *S. carpenteri* vs. *S. chacei*; *S. kensleyi* vs. *S. microneptunus*), and one pair of eusocial species (EE: *S. filidigitus* vs. *S. regalis*). Then, we used a linear model to test whether d*N*, d*S*, and ω increased or decreased along a discrete gradient of social groups (NN–NE–EE), using *R*. In treating social groups as an ordinal factor, we expected eusocial-specific substitution rates to have no effect in the NN group, some effects in the NE group, and a strong effect in the EE group. In addition to the factor social groups, our linear model also controlled for the difference in length of PCGs and the effect of genetic distance. Preliminary analyses showed that there was no interaction between social groups and PCGs in predicting d*N*, d*S*, and ω, but there is variation among PCGs, particularly related to the length of each gene. Correlation between gene length and substitution rate has been found elsewhere (Eyre-Walker 1996); therefore, we used gene length to control for the length variability among PCGs. Further, as the branch lengths between each pair of *Synalpheus* species were not equal, our linear model included the effect of genetic distance between pairs of species, using the pairwise branch length between *Synalpheus* species from our mitochondrial-based phylogenies. Genetic distances were obtained using the *R* package *ape* (Paradis et al. 2004). The pairwise distances were not identical between the nucleotide and amino acid trees (adj. *R*^2^ = 0.817 and 0.852, for BI and ML respectively). However, the results were identical regardless of the distances used, so we presented only data using the nucleotide distance.

As a supplement, we also analyzed all pairs between each *Synalpheus* species and an *Alpheus* species (40 *Synalpheus–Alpheus* pairs). We found that the estimated substitution rates were strongly influenced by the choice of *Alpheus* species in the pairwise comparison. Therefore, we controlled for this heterogeneity through random factor and outlier-removal approaches (see Supplementary Material online).

### Within-Gene Comparison among *Synalpheus* Sister Pairs

We employed a sliding-windows approach to compare d*N*, d*S*, and ω between short sections within each PCG (57 bp long and 6 bp apart) among species pairs. For *Synalpheus* sister pairs, we performed the same ordinal factor linear regression as above.

### Signs of Selection: PAML and Secondary Structure Predictions

First, we employed “branch models” in *PAML* (Yang 1997, 2007) via *pamlX* (Xu and Yang 2013) to test whether eusocial branches have different ω values for each PCGs. We ran a free-ratio model in which ω values were free to vary in each branch and the two-ratio model in which the foreground (eusocial branches) had a different ω value from the background (all other branches). These models were compared with the fixed ratio model in which the ω value was fixed to have the same value across branches using the test statistic as two times the difference in log-likelihood and the chi-square distribution. From the free-ratio model, we tested whether d*N*, d*S*, and ω differed between terminal branches leading to eusocial versus noneusocial species across all PCGs using an ANOVA, controlling for the effect of PCG length. The genes *nad4l* in *S. chacei* and *S. filidigitus* and *atp8* in *S. kensleyi* had zero d*N*, so we excluded them from analysis of d*N* and ω.

Then, we ran a “branch-site test of positive selection” (Yang 2005; Zhang et al. 2005) to test whether a proportion of codons in eusocial lineages (foreground) underwent positive selection compared with those in noneusocial lineages (background) for each PCG. We ran the alternative model (model A) which estimates the proportions and ω in four classes among sites: class 0, 0 < ω_0_ < 1; class 1, ω_1_ = 1; class 2a, 0 < ω_0_ < 1 for the background and ω_2_ ≥ 1 for the foreground; class 2b, ω_1_ = 1 for the background and ω_2_ ≥  for the foreground. Model A was compared with a null model in which ω_2_ = 1. From Model A, sites with positive selection only in eusocial lineages were identified with BEB inference (Yang 2005). The results were identical regardless of the use of nucleotide or amino acid ML trees to provide initial branch length to *PAML*; we therefore presented only results based on the nucleotide ML tree.

To examine the changes in the positively selected sites, we predicted the secondary structures and relative accessible surface area (rSA) of protein for each PCG using the *Predict Protein* webserver (Yachdav et al. 2014). Each amino acid was predicted to form either an a-helix (H), b-sheet (S), or w-loop (L), each with a reliability index (0 = low, 9 = high). Also, the relative solvent accessibility was coded as buried (b, rSA = 0–9%), intermediate (i, rSA = 9–36%), and exposed (e, rSA = 36–100%). For sister *Synalpheus* species that had different amino acids at positively selected sites, we used *SNAP2* (Hecht et al. 2015) implemented in the *Predict Protein* webserver to predict whether the change would affect protein function (as neutral or effect changes), indicative of adaptive changes.

### Signs of Selection: *HyPhy*

Further, we used several methods available in *HyPhy* (Kosakovsky Pond et al. 2020) to test, among the eight *Synalpheus* species, whether branches leading to eusocial species show signs of positive selection or relaxed purifying selection. We tested for signs of positive selection at branches leading to eusocial species. We used the *aBSREL* (adaptive branch-site random effects likelihood) method which assumes that eusocial branches may be subject to episodic positive selection at a proportion of sites (Smith et al. 2015). This is an improved version of the “branch-site” models in *PAML*. As our data set is small, it may not have enough power to detect positive selection. Therefore, we used the *BUSTED* (branch-site unrestricted statistical test for episodic diversification) method to test for positive selection of a gene at any site on eusocial branches (at least one site on at least one branch) (Murrell et al. 2015).

Alternatively, eusocial species may show relaxed purifying selection at mitochondrial PCGs. We used the *RELAX* (Wertheim et al. 2015) method to test for a relaxation of selection pressure along eusocial branches. Signatures of positive selection can resemble relaxed purifying selection. The program *RELAX* distinguishes between these signals by modeling how codons with different ω categories (ω > 1 and ω < 1) respond to a single selection intensity parameter *k*. Relaxed selection would push all ω categories toward 1. The parameter *k* modifies ω (as ω^*k*^) at terminal branches leading to eusocial species compared with branches leading to noneusocial species. *k *>* *1 indicates an increased selection strength, whereas *k *<* *1 indicates a relaxed selection strength at eusocial branches. *RELAX* uses a log-likelihood ratio test to compare the supports for the null model (*k *=* *1) and the alternative model (*k *>* *1 or *k *<* *1) (Wertheim et al. 2015).

## Supplementary Material

Supplementary data are available at *Molecular Biology and Evolution* online.

## Supplementary Material

msaa297_Supplementary_Data
