# Supplementary material for: Eusociality Shapes Convergent Patterns of Molecular Evolution across Mitochondrial Genomes of Snapping Shrimps
Source: Mol Biol Evol. 2020 Nov 19;38(4):1372–83. doi: 10.1093/molbev/msaa297 (PMC8480187; doi:10.1093/molbev/msaa297)
Supplement: msaa297_Supplementary_Data [file msaa297_Supplementary_Data.zip › mt_comp_sup_20200728_MBE_submitted.pdf]

# Supplementary Material

## 1. Supplementary Methods and Results

### Methods

*Bayesian phylogenetic inference*

We optimized matrices under BI in MrBayes (Huelsenbeck and Ronquist 2001). We used ModelTest-NG (Darriba, et al. 2019) to identify appropriate models for each partition. Our MrBayes tree searches employed two simultaneous runs, each with four chains set to default temperature settings. The analysis ran for 5,000,000 cycles with 25 % of samples discarded as burn-in and sampling frequency of 500. We checked for convergence using the standard deviation of split frequencies (< 0.01) and by assessing the effective sample size (>100) and potential scale reduction factor (close to 1).

*Pairwise $K_A/K_S$ in Synalpheus-Alpheus pairs*

We analyzed all pairs between each *Synalpheus* species and an *Alpheus* species (*A. bellulus, A. distinguendus, A. inopinatus, A. lobindens,* and *A. randalli*) (40 *Synalpheus-Alpheus* pairs). These pairs can be grouped into two social groups: either a eusocial *Synalpheus* paired with an *Alpheus* species (NE) or a non-eusocial *Synalpheus* species paired with an *Alpheus* species (NN). Because each *Alpheus* species has its own evolutionary history, the values of dN, dS, and ω between a *Synalpheus* species and each of the five *Alpheus* species cannot be assumed to be equal. Indeed, we found that for each *Synalpheus* species, the values of  dN, dS, and ω varied, often drastically, dependent on which *Alpheus* species were used (supplementary fig. S1). These outliers likely reflect species-specific substitution rate shifts in specific *Alpheus* lineages. We used two methods to mitigate this problem.

First, we treated *Alpheus* as a random factor and used phylogenetic mixed models implemented in *MCMCglmm* v2.29 (Hadfield 2010) to test the effect of social groups on dN, dS, and ω for each PCGs and the concatenated PCGs. We used weakly informative priors (Variance parameters, V = 1, degree of belief, nu = 0.002) and ran 2,000,000 Markov Chain Monte Carlo (MCMC) iterations with 50,000 iterations of burn-in and a thinning interval of 250.

Alternatively, for each *Synalpheus* species, we removed outlier values of dN, dS, and ω across *Alpheus* species (if the values were 1.5 interquartile ranges below the first quartile or above the third quartile), then we took the mean of the remaining values to represent the dN, dS, and ω between a *Synalpheus* species and the *Alpheus* outgroup. Preliminary analyses showed that there was no interaction effect between *Alpheus* species and social groups in predicting dN, dS, and ω, except for cox1 and dS,

which we analyzed separately for each *Alpheus* species. Then, for each PCGs and the concatenated PCGs, we used phylogenetic least squared regressions to test whether the mean values of dN, dS, and ω differed between social groups (NE vs. NN), controlling for the phylogenetic independence between species data. We used a published *Synalpheus* phylogeny trimmed to relevant taxa (Chak, et al. 2017) and used both BM and OU models of trait evolution. Further, we used *MCMCglmm* to test the effect of social groups across PCGs using the mean dN, dS, and ω after outlier removal.

Along sliding windows across PCGs, we used only the phylogenetic mixed model that treated *Alpheus* as a random factor.

**Results**

*Bayesian phylogenetic inference*

Bayesian phylogenetic trees are shown in supplementary fig. S1 and their topology is similar to that of the maximum likelihood trees based on nucleotides and amino acids, respectively.

*Pairwise $K_A/K_S$ in Synalpheus-Alpheus pairs*

Across *Synalpheus-Alpheus* pairs, dN, dS, and ω did not differ between eusocial and non-eusocial species in all PCGs and the concatenated PCG when *Alpheus* species were treated as a random factor using phylogenetic mixed models. Similarly, after outlier removal, mean values of dN, dS, and ω did not differ between eusocial and non-eusocial species in all PCGs and the concatenated PCG, with a few exceptions (supplementary fig. S1). First, values of dN were significantly higher in eusocial species in *cox1* (BM: $p = 0.003$, OU: $p = 0.015$) and *apt6* (BM: $p = 0.025$, OU: $p = 0.026$), and marginally lower in eusocial species in *atp8* (BM: $p = 0.052$, OU: $p = 0.049$). Second, the use of *Alpheus* outgroup species significantly affected how dS was predicted by social group in *cox1*. While dS was lower in eusocial *Synalpheus* species when calculated against four *Alpheus* species (*A. bellulus, A. distinguendus, A. inopinatus,* and *A. lobindens*), it was higher in eusocial species when calculated against *S. randalli*. Incorporating the variation across PCGs using *MCMCglmm*, there was no significant effect of the factor social groups in predicting dN, dS, and ω. Despite lacking statistical significance, the trends observed among *Synalpheus-Alpheus* pairs were similar within-*Synalpheus* comparisons: eusocial lineages have the tendency to have higher ω and dN and lower dS.

**References**

Chak STC, Duffy JE, Hultgren KM, Rubenstein DR. 2017. Evolutionary transitions towards eusociality in snapping shrimps. Nature Ecology & Evolution 1:0096.

Darriba D, Posada D, Kozlov AM, Stamatakis A, Morel B, Flouri T. 2019. ModelTest-NG: a new and scalable tool for the selection of DNA and protein evolutionary models. Molecular Biology and Evolution.

Hadfield JD. 2010. MCMC methods for multi-response generalized linear mixed models: the MCMCglmm R package. Journal of statistical software 33:1-22.

Huelsenbeck JP, Ronquist F. 2001. MRBAYES: Bayesian inference of phylogenetic trees. Bioinformatics 17:754-755.

## 2. Supplementary Figures

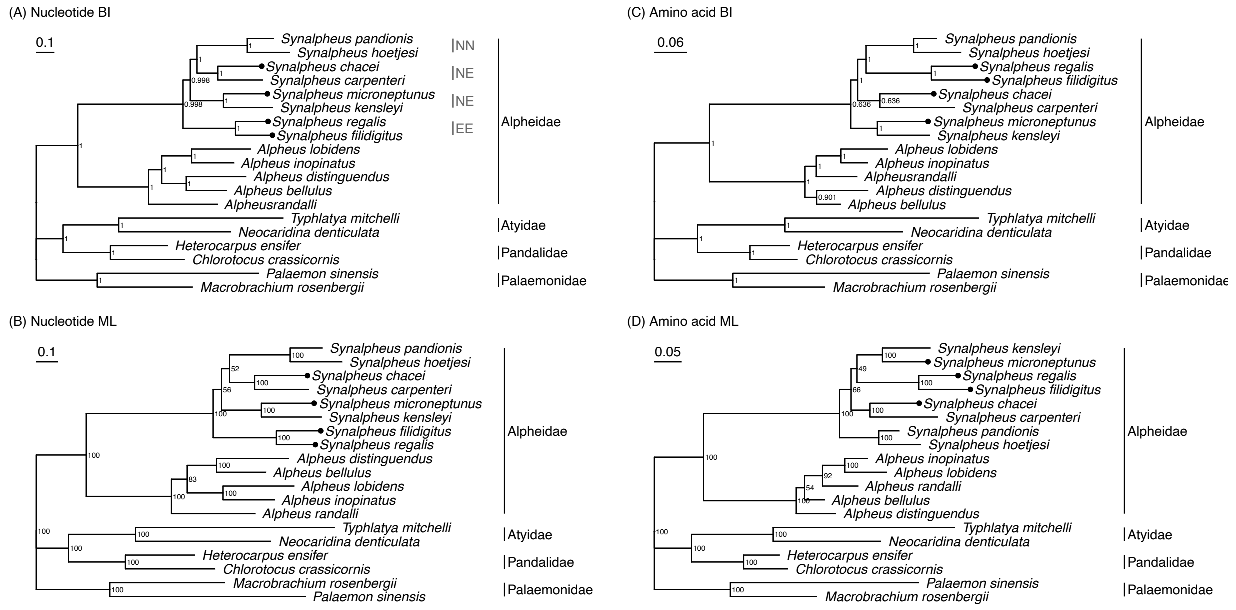


FIG. S1. Phylogenetic trees based on 13 PCGs. (A) nucleotide ML tree, (B) nucleotide BI tree, (C) amino acid ML tree, and (D) amino acid BI tree. Node values are bootstrap parentages in ML trees and posterior probability in BI trees. Black vertical bars and labels indicate the four Caridean families. Solid dots at terminal branches indicate eusocial species. Grey vertical bars and labels in (A) indicate the social groups used in the pairwise analyses.

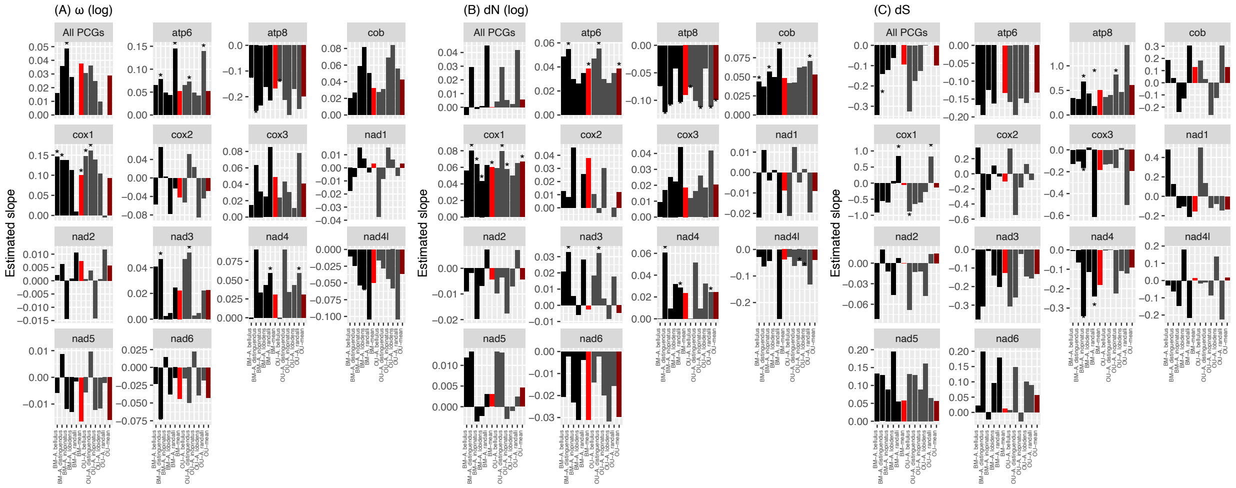


FIG. S2. Regression coefficients (slope or b) of the effect of sociality (eusocial vs. non-eusocial, +ve slope indicates that eusocial species have higher values) on dN, dS, and ω when *Synalpheus* species were compared to different *Alpheus* outgroup species and predicted using the BM and OU models of trait evolution. Outliers values of dN, dS, and ω across *Alpheus* species were removed. Red and dark red bars represent analyses where the mean values of dN, dS, and ω were used, after removing outlier values across *Alpheus* species for BM and OU, respectively. Asterisks represent significant effects of sociality.

## 3. Supplementary Tables

Table S1. Samples used for mitogenome assembly. NA: Mitochondrial genomes were manually circularized and thus no information on chromosome coverage was made available from the assembly software *NOVOPlasty*.

| *Synalpheus sp.* | Voucher # | Collection year | Collection country | SRA | Pair-end sequences (G bases) | Assembly coverage | Assembly size (bp) | Assembly accession |
|---|---|---|---|---|---|---|---|---|
| *pandionis* | JAM2008-090-001 | 2008 | Jamaica | SRX6711387 | 8.6 | 53 | 15,465 | MN787593 |
| *carpenteri* | JAM2008-026-001 | 2008 | Jamaica | SRX6711392 | 13.3 | 155 | 15,594 | MN787594 |
| *kensleyi* | CBC2012-023-001 | 2012 | Belize | SRX6711386 | 12.5 | 44 | 15,638 | MN787595 |
| *chacei* | CBC2004-061-002 | 2004 | Belize | SRX6711391 | 11.2 | 90 | 15,469 | MN787596 |
| *hoetjesi* | CU2008-003-003 | 2008 | Curacao | SRX6711389 | 6.9 | NA | 15,421 | MN787597 |
| *regalis* | JAM2008-063-001 | 2008 | Jamaica | SRX6711385 | 18.6 | 77 | 15,658 | MN787598 |
| *filidigitus* | CBC2009_077_002 | 2009 | Belize | SRX6711390 | 14 | NA | 15,782 | MN787599 |

Table S2. Mitogenome annotations of seven *Synalpheus* species.

| *Synalpheus sp.* | Name | Type | Start | Stop | Strand | Length (bp) | Start | Stop | Intergenic space |
|---|---|---|---|---|---|---|---|---|---|
| *carpenteri* | Cox1 | Coding | 1 | 1539 | + | 1539 | CGA | TAA | -5 |
| | trnL2(tta) | tRNA | 1535 | 1600 | + | 66 | | | 16 |
| | cox2 | Coding | 1617 | 2324 | + | 708 | ATG | TAA | -20 |
| | trnK(aaa) | tRNA | 2305 | 2373 | + | 69 | | | 2 |
| | trnD(gac) | tRNA | 2376 | 2440 | + | 65 | | | 11 |
| | atp8 | Coding | 2452 | 2602 | + | 151 | ATA | T | -13 |
| | atp6 | Coding | 2590 | 3264 | + | 675 | ATG | TAA | -4 |
| | cox3 | Coding | 3261 | 4054 | + | 794 | ATA | T | -4 |
| | trnG(gga) | tRNA | 4051 | 4119 | + | 69 | | | 6 |
| | nad3 | Coding | 4126 | 4473 | + | 348 | ATA | TAA | 0 |
| | trnA(gca) | tRNA | 4474 | 4536 | + | 63 | | | 1 |
| | trnR(cga) | tRNA | 4538 | 4602 | + | 65 | | | -2 |
| | trnN(aac) | tRNA | 4601 | 4666 | + | 66 | | | 0 |
| | trnS1(aga) | tRNA | 4667 | 4724 | + | 58 | | | 8 |
| | trnE(gaa) | tRNA | 4733 | 4798 | + | 66 | | | 0 |
| | trnF(ttc) | tRNA | 4799 | 4864 | - | 66 | | | 0 |
| | nad5 | Coding | 4865 | 6565 | - | 1701 | ATA | TAG | 15 |
| | trnH(cac) | tRNA | 6581 | 6643 | - | 63 | | | -1 |
| | nad4 | Coding | 6643 | 7980 | - | 1338 | ATA | TAG | -4 |
| | nad4l | Coding | 7977 | 8270 | - | 294 | ATG | TAA | 8 |
| | trnT(aca) | tRNA | 8279 | 8339 | + | 61 | | | 0 |
| | trnP(cca) | tRNA | 8340 | 8403 | - | 64 | | | 11 |
| | nad6 | Coding | 8415 | 8921 | + | 507 | ATA | TAA | -1 |
| | cob | Coding | 8921 | 10055 | + | 1135 | ATG | T | 0 |
| | trnS2(tca) | tRNA | 10056 | 10125 | + | 70 | | | -1 |
| | nad1 | Coding | 10125 | 11072 | - | 948 | ATA | TAA | 25 |
| | trnL1(cta) | tRNA | 11098 | 11164 | - | 67 | | | -49 |
| | rrnL | rRNA | 11116 | 12479 | - | 1364 | - | - | -8 |
| | trnV(gta) | tRNA | 12472 | 12536 | - | 65 | | | -1 |
| | rrnS | rRNA | 12536 | 13328 | - | 793 | - | - | 0 |
| | CR Putative | | 13329 | 14199 | | 871 | | | 0 |
| | trnI(atc) | tRNA | 14200 | 14264 | + | 65 | | | 4 |
| | trnQ(caa) | tRNA | 14269 | 14336 | - | 68 | | | -1 |
| | trnM(atg) | tRNA | 14336 | 14401 | + | 66 | | | 24 |
| | nad2 | Coding | 14426 | 15403 | + | 978 | ATA | TAA | -2 |
| | trnW(tga) | tRNA | 15402 | 15466 | + | 65 | | | -1 |
| | trnC(tgc) | tRNA | 15466 | 15527 | - | 62 | | | 1 |
| | trnY(tac) | tRNA | 15529 | 15594 | - | 66 | | | |
| *chacei* | Cox1 | Coding | 1 | 1539 | + | 1539 | ATG | TAA | -5 |
| | trnL2(tta) | tRNA | 1535 | 1601 | + | 67 | | | 20 |
| | cox2 | Coding | 1622 | 2329 | + | 708 | ATG | TAA | -20 |
| | trnK(aaa) | tRNA | 2310 | 2378 | + | 69 | | | 2 |
| | trnD(gac) | tRNA | 2381 | 2443 | + | 63 | | | 0 |

| | | | | | | | | | |
|---|---|---|---|---|---|---|---|---|---|
| | atp8 | Coding | 2444 | 2599 | + | 156 | ATG | TAG | -7 |
| | atp6 | Coding | 2593 | 3267 | + | 675 | ATG | TAA | -1 |
| | cox3 | Coding | 3267 | 4070 | + | 804 | ATG | TAA | -17 |
| | trnG(gga) | tRNA | 4054 | 4118 | + | 65 | | | 6 |
| | nad3 | Coding | 4125 | 4472 | + | 348 | ATA | TAA | 0 |
| | trnA(gca) | tRNA | 4473 | 4535 | + | 63 | | | 1 |
| | trnR(cga) | tRNA | 4537 | 4601 | + | 65 | | | -2 |
| | trnN(aac) | tRNA | 4600 | 4665 | + | 66 | | | 0 |
| | trnS1(aga) | tRNA | 4666 | 4723 | + | 58 | | | 5 |
| | trnE(gaa) | tRNA | 4729 | 4794 | + | 66 | | | 0 |
| | trnF(ttc) | tRNA | 4795 | 4858 | - | 64 | | | 0 |
| | nad5 | Coding | 4859 | 6556 | - | 1698 | ATG | TAA | 18 |
| | trnH(cac) | tRNA | 6575 | 6638 | - | 64 | | | -1 |
| | nad4 | Coding | 6638 | 7975 | - | 1338 | ATG | TAG | -4 |
| | nad4l | Coding | 7972 | 8265 | - | 294 | ATG | TAA | 2 |
| | trnT(aca) | tRNA | 8268 | 8329 | + | 62 | | | 0 |
| | trnP(cca) | tRNA | 8330 | 8394 | - | 65 | | | -16 |
| | nad6 | Coding | 8379 | 8912 | + | 534 | ATT | TAA | -1 |
| | cob | Coding | 8912 | 10049 | + | 1138 | ATG | T | -3 |
| | trnS2(tca) | tRNA | 10047 | 10116 | + | 70 | | | 22 |
| | nad1 | Coding | 10139 | 11095 | - | 957 | ATA | TAA | -3 |
| | trnL1(cta) | tRNA | 11093 | 11157 | - | 65 | | | -44 |
| | rrnL | rRNA | 11114 | 12489 | - | 1376 | - | - | -18 |
| | trnV(gta) | tRNA | 12472 | 12536 | - | 65 | | | -1 |
| | rrnS | rRNA | 12536 | 13333 | - | 798 | - | - | 0 |
| | CR [Putative] | | 13334 | 14068 | | 735 | | | 0 |
| | trnI(atc) | tRNA | 14069 | 14132 | + | 64 | | | 7 |
| | trnQ(caa) | tRNA | 14140 | 14208 | - | 69 | | | -1 |
| | trnM(atg) | tRNA | 14208 | 14271 | + | 64 | | | -21 |
| | nad2 | Coding | 14251 | 15273 | + | 1023 | ATG | TAA | -2 |
| | trnW(tga) | tRNA | 15272 | 15336 | + | 65 | | | -1 |
| | trnC(tgc) | tRNA | 15336 | 15401 | - | 66 | | | 2 |
| | trnY(tac) | tRNA | 15404 | 15469 | - | 66 | | | |
| *filidigitus* | Cox1 | Coding | 1 | 1539 | + | 1539 | ACG | TAA | -5 |
| | trnL2(tta) | tRNA | 1535 | 1601 | + | 67 | | | 14 |
| | cox2 | Coding | 1616 | 2323 | + | 708 | ATG | TAA | -20 |
| | trnK(aaa) | tRNA | 2304 | 2373 | + | 70 | | | 2 |
| | trnD(gac) | tRNA | 2376 | 2438 | + | 63 | | | 9 |
| | atp8 | Coding | 2448 | 2594 | + | 147 | ATG | TAA | -7 |
| | atp6 | Coding | 2588 | 3265 | + | 678 | ATG | TAA | 4 |
| | cox3 | Coding | 3270 | 4056 | + | 787 | ATG | T | 0 |
| | trnG(gga) | tRNA | 4057 | 4120 | + | 64 | | | 6 |
| | nad3 | Coding | 4127 | 4474 | + | 348 | ATA | TAA | -1 |
| | trnA(gca) | tRNA | 4474 | 4534 | + | 61 | | | 1 |
| | trnR(cga) | tRNA | 4536 | 4601 | + | 66 | | | -2 |
| | trnN(aac) | tRNA | 4600 | 4669 | + | 70 | | | 0 |

| | | | | | | | | | |
|---|---|---|---|---|---|---|---|---|---|
| | trnS1(aga) | tRNA | 4670 | 4726 | + | 57 | | | 9 |
| | trnE(gaa) | tRNA | 4736 | 4801 | + | 66 | | | 0 |
| | trnF(ttc) | tRNA | 4802 | 4869 | - | 68 | | | -1 |
| | nad5 | Coding | 4869 | 6572 | - | 1704 | ATA | TAG | 18 |
| | trnH(cac) | tRNA | 6591 | 6658 | - | 68 | | | -1 |
| | nad4 | Coding | 6658 | 7995 | - | 1338 | ATA | TAG | -4 |
| | nad4l | Coding | 7992 | 8285 | - | 294 | ATG | TAA | 2 |
| | trnT(aca) | tRNA | 8288 | 8349 | + | 62 | | | 0 |
| | trnP(cca) | tRNA | 8350 | 8415 | - | 66 | | | 17 |
| | nad6 | Coding | 8433 | 8933 | + | 501 | ATA | TAA | -1 |
| | cob | Coding | 8933 | 10075 | + | 1143 | ATG | TAA | -8 |
| | trnS2(tca) | tRNA | 10068 | 10137 | + | 70 | | | -2 |
| | nad1 | Coding | 10136 | 11140 | - | 1005 | ATA | TAG | -28 |
| | trnL1(cta) | tRNA | 11113 | 11179 | - | 67 | | | -43 |
| | rrnL | rRNA | 11137 | 12501 | - | 1365 | - | - | -6 |
| | trnV(gta) | tRNA | 12496 | 12559 | - | 64 | | | -1 |
| | rrnS | rRNA | 12559 | 13365 | - | 807 | - | - | 0 |
| | CR Putative | | 13366 | 14359 | | 994 | | | 0 |
| | trnI(atc) | tRNA | 14360 | 14423 | + | 64 | | | 15 |
| | trnQ(caa) | tRNA | 14439 | 14507 | - | 69 | | | -2 |
| | trnM(atg) | tRNA | 14506 | 14571 | + | 66 | | | 27 |
| | nad2 | Coding | 14599 | 15573 | + | 975 | ATA | TAG | -1 |
| | trnW(tga) | tRNA | 15573 | 15636 | + | 64 | | | 15 |
| | trnC(tgc) | tRNA | 15652 | 15715 | - | 64 | | | 2 |
| | trnY(tac) | tRNA | 15718 | 15782 | - | 65 | | | |
| *hoetjesi* | Cox1 | Coding | 1 | 1539 | + | 1539 | ATG | TAA | -5 |
| | trnL2(tta) | tRNA | 1535 | 1601 | + | 67 | | | 18 |
| | cox2 | Coding | 1620 | 2327 | + | 708 | ATG | TAA | -20 |
| | trnK(aaa) | tRNA | 2308 | 2376 | + | 69 | | | 2 |
| | trnD(gac) | tRNA | 2379 | 2440 | + | 62 | | | 9 |
| | atp8 | Coding | 2450 | 2596 | + | 147 | ATA | TAG | -7 |
| | atp6 | Coding | 2590 | 3264 | + | 675 | ATG | TAA | -4 |
| | cox3 | Coding | 3261 | 4071 | + | 811 | ATA | TAG | -21 |
| | trnG(gga) | tRNA | 4051 | 4117 | + | 67 | | | 9 |
| | nad3 | Coding | 4127 | 4471 | + | 345 | ATA | TAA | 1 |
| | trnA(gca) | tRNA | 4473 | 4535 | + | 63 | | | 7 |
| | trnR(cga) | tRNA | 4543 | 4605 | + | 63 | | | -2 |
| | trnN(aac) | tRNA | 4604 | 4669 | + | 66 | | | 0 |
| | trnS1(aga) | tRNA | 4670 | 4726 | + | 57 | | | 32 |
| | trnE(gaa) | tRNA | 4759 | 4823 | + | 65 | | | 3 |
| | trnF(ttc) | tRNA | 4827 | 4891 | - | 65 | | | -1 |
| | nad5 | Coding | 4891 | 6606 | - | 1716 | ATG | TAA | 0 |
| | trnH(cac) | tRNA | 6607 | 6671 | - | 65 | | | -1 |
| | nad4 | Coding | 6671 | 8008 | - | 1338 | ATA | TAG | -4 |
| | nad4l | Coding | 8005 | 8298 | - | 294 | ATG | TAA | 1 |
| | trnT(aca) | tRNA | 8300 | 8361 | + | 62 | | | 0 |

| | | | | | | | | |
|---|---|---|---|---|---|---|---|---|
| | trnP(cca) | tRNA | 8362 | 8425 | - | 64 | | | 2 |
| | nad6 | Coding | 8428 | 8943 | + | 516 | ATT | TAA | -1 |
| | cob | Coding | 8943 | 10077 | + | 1135 | ATG | T | 0 |
| | trnS2(tca) | tRNA | 10078 | 10147 | + | 70 | | | 22 |
| | nad1 | Coding | 10170 | 11099 | - | 930 | ATA | TAA | 25 |
| | trnL1(cta) | tRNA | 11125 | 11189 | - | 65 | | | -56 |
| | rrnL | rRNA | 11134 | 12505 | - | 1372 | - | - | -18 |
| | trnV(gta) | tRNA | 12488 | 12550 | - | 63 | | | 0 |
| | rrnS | rRNA | 12551 | 13364 | - | 814 | - | - | 0 |
| | CR Putative | | 13365 | 14012 | | 648 | | | 0 |
| | trnI(atc) | tRNA | 14013 | 14076 | + | 64 | | | 17 |
| | trnQ(caa) | tRNA | 14094 | 14161 | - | 68 | | | -1 |
| | trnM(atg) | tRNA | 14161 | 14226 | + | 66 | | | 27 |
| | nad2 | Coding | 14254 | 15228 | + | 975 | ATA | TAA | -1 |
| | trnW(tga) | tRNA | 15228 | 15291 | + | 64 | | | -1 |
| | trnC(tgc) | tRNA | 15291 | 15353 | - | 63 | | | 2 |
| | trnY(tac) | tRNA | 15356 | 15421 | - | 66 | | | |
| *kensleyi* | Cox1 | Coding | 1 | 1539 | + | 1539 | ACG | TAA | -5 |
| | trnL2(tta) | tRNA | 1535 | 1601 | + | 67 | | | 15 |
| | cox2 | Coding | 1617 | 2295 | + | 679 | ATA | T | 15 |
| | trnK(aaa) | tRNA | 2311 | 2379 | + | 69 | | | 3 |
| | trnD(gac) | tRNA | 2383 | 2445 | + | 63 | | | 9 |
| | atp8 | Coding | 2455 | 2608 | + | 154 | ATA | T | -14 |
| | atp6 | Coding | 2595 | 3269 | + | 675 | ATG | TAA | -4 |
| | cox3 | Coding | 3266 | 4055 | + | 790 | ATA | T | 0 |
| | trnG(gga) | tRNA | 4056 | 4121 | + | 66 | | | 6 |
| | nad3 | Coding | 4128 | 4475 | + | 348 | ATA | TAG | -1 |
| | trnA(gca) | tRNA | 4475 | 4537 | + | 63 | | | 5 |
| | trnR(cga) | tRNA | 4543 | 4608 | + | 66 | | | -2 |
| | trnN(aac) | tRNA | 4607 | 4670 | + | 64 | | | 0 |
| | trnS1(aga) | tRNA | 4671 | 4727 | + | 57 | | | 14 |
| | trnE(gaa) | tRNA | 4742 | 4807 | + | 66 | | | 1 |
| | trnF(ttc) | tRNA | 4809 | 4873 | - | 65 | | | 1 |
| | nad5 | Coding | 4875 | 6572 | - | 1698 | ATA | TAA | 18 |
| | trnH(cac) | tRNA | 6591 | 6654 | - | 64 | | | -1 |
| | nad4 | Coding | 6654 | 7991 | - | 1338 | ATA | TAG | -4 |
| | nad4l | Coding | 7988 | 8281 | - | 294 | ATG | TAA | 1 |
| | trnT(aca) | tRNA | 8283 | 8343 | + | 61 | | | 0 |
| | trnP(cca) | tRNA | 8344 | 8408 | - | 65 | | | 2 |
| | nad6 | Coding | 8411 | 8926 | + | 516 | ATA | TAA | -1 |
| | cob | Coding | 8926 | 10060 | + | 1135 | ATG | T | 0 |
| | trnS2(tca) | tRNA | 10061 | 10130 | + | 70 | | | -2 |
| | nad1 | Coding | 10129 | 11082 | - | 954 | TAG | TAG | 25 |
| | trnL1(cta) | tRNA | 11108 | 11172 | - | 65 | | | -42 |
| | rrnL | rRNA | 11131 | 12496 | - | 1366 | - | - | -17 |
| | trnV(gta) | tRNA | 12480 | 12545 | - | 66 | | | 0 |

| | | | | | | | | | |
|---|---|---|---|---|---|---|---|---|---|
| | rrnS | rRNA | 12546 | 13343 | - | 798 | - | - | 0 |
| | CR Putative | | 13344 | 14222 | | 879 | | | 0 |
| | trnI(atc) | tRNA | 14223 | 14286 | + | 64 | | | 18 |
| | trnQ(caa) | tRNA | 14305 | 14373 | - | 69 | | | -1 |
| | trnM(atg) | tRNA | 14373 | 14438 | + | 66 | | | 27 |
| | nad2 | Coding | 14466 | 15440 | + | 975 | ATA | TAA | -2 |
| | trnW(tga) | tRNA | 15439 | 15503 | + | 65 | | | -1 |
| | trnC(tgc) | tRNA | 15503 | 15568 | - | 66 | | | 1 |
| | trnY(tac) | tRNA | 15570 | 15638 | - | 69 | | | |
| *pandionis* | Cox1 | Coding | 1 | 1539 | + | 1539 | CGA | TAA | -5 |
| | trnL2(tta) | tRNA | 1535 | 1601 | + | 67 | | | 17 |
| | cox2 | Coding | 1619 | 2327 | + | 709 | ATG | TAG | -21 |
| | trnK(aaa) | tRNA | 2307 | 2375 | + | 69 | | | 2 |
| | trnD(gac) | tRNA | 2378 | 2440 | + | 63 | | | 9 |
| | atp8 | Coding | 2450 | 2596 | + | 147 | ATA | TAG | -7 |
| | atp6 | Coding | 2590 | 3264 | + | 675 | ATG | TAA | -4 |
| | cox3 | Coding | 3261 | 4050 | + | 790 | ATA | T | 0 |
| | trnG(gga) | tRNA | 4051 | 4117 | + | 67 | | | 6 |
| | nad3 | Coding | 4124 | 4471 | + | 348 | ATA | TAA | 1 |
| | trnA(gca) | tRNA | 4473 | 4535 | + | 63 | | | 8 |
| | trnR(cga) | tRNA | 4544 | 4605 | + | 62 | | | -2 |
| | trnN(aac) | tRNA | 4604 | 4669 | + | 66 | | | 0 |
| | trnS1(aga) | tRNA | 4670 | 4727 | + | 58 | | | 10 |
| | trnE(gaa) | tRNA | 4738 | 4803 | + | 66 | | | 3 |
| | trnF(ttc) | tRNA | 4807 | 4870 | - | 64 | | | -1 |
| | nad5 | Coding | 4870 | 6585 | - | 1716 | ATG | TAA | 0 |
| | trnH(cac) | tRNA | 6586 | 6648 | - | 63 | | | 0 |
| | nad4 | Coding | 6649 | 7986 | - | 1338 | ATA | TAA | -4 |
| | nad4l | Coding | 7983 | 8276 | - | 294 | ATG | TAA | 1 |
| | trnT(aca) | tRNA | 8278 | 8338 | + | 61 | | | 0 |
| | trnP(cca) | tRNA | 8339 | 8402 | - | 64 | | | 8 |
| | nad6 | Coding | 8411 | 8920 | + | 510 | ATA | TAA | -1 |
| | cob | Coding | 8920 | 10057 | + | 1138 | ATG | T | -3 |
| | trnS2(tca) | tRNA | 10055 | 10124 | + | 70 | | | -4 |
| | nad1 | Coding | 10121 | 11114 | - | 994 | ATA | T | -16 |
| | trnL1(cta) | tRNA | 11099 | 11163 | - | 65 | | | -50 |
| | rrnL | rRNA | 11114 | 12494 | - | 1381 | - | - | -23 |
| | trnV(gta) | tRNA | 12472 | 12534 | - | 63 | | | 0 |
| | rrnS | rRNA | 12535 | 13343 | - | 809 | - | - | 0 |
| | CR Putative | | 13344 | 14052 | | 709 | | | 0 |
| | trnI(atc) | tRNA | 14053 | 14116 | + | 64 | | | 18 |
| | trnQ(caa) | tRNA | 14135 | 14202 | - | 68 | | | -1 |
| | trnM(atg) | tRNA | 14202 | 14268 | + | 67 | | | 27 |
| | nad2 | Coding | 14296 | 15270 | + | 975 | ATA | TAA | -1 |
| | trnW(tga) | tRNA | 15270 | 15333 | + | 64 | | | 0 |
| | trnC(tgc) | tRNA | 15334 | 15397 | - | 64 | | | 2 |

| | | | | | | | | | |
|---|---|---|---|---|---|---|---|---|---|
| | trnY(tac) | tRNA | 15400 | 15465 | - | 66 | | | |
| *regalis* | Cox1 | Coding | 1 | 1539 | + | 1539 | ACG | TAA | -5 |
| | trnL2(tta) | tRNA | 1535 | 1601 | + | 67 | | | 14 |
| | cox2 | Coding | 1616 | 2326 | + | 711 | ATG | TAA | -20 |
| | trnK(aaa) | tRNA | 2307 | 2376 | + | 70 | | | 2 |
| | trnD(gac) | tRNA | 2379 | 2441 | + | 63 | | | 9 |
| | atp8 | Coding | 2451 | 2597 | + | 147 | ATA | TAG | -7 |
| | atp6 | Coding | 2591 | 3268 | + | 678 | ATG | TAA | 0 |
| | cox3 | Coding | 3269 | 4055 | + | 787 | ATG | T | 0 |
| | trnG(gga) | tRNA | 4056 | 4120 | + | 65 | | | 6 |
| | nad3 | Coding | 4127 | 4474 | + | 348 | ATA | TAA | -2 |
| | trnA(gca) | tRNA | 4473 | 4537 | + | 65 | | | 0 |
| | trnR(cga) | tRNA | 4538 | 4601 | + | 64 | | | -2 |
| | trnN(aac) | tRNA | 4600 | 4667 | + | 68 | | | 0 |
| | trnS1(aga) | tRNA | 4668 | 4724 | + | 57 | | | 10 |
| | trnE(gaa) | tRNA | 4735 | 4800 | + | 66 | | | 25 |
| | trnF(ttc) | tRNA | 4826 | 4889 | - | 64 | | | 8 |
| | nad5 | Coding | 4898 | 6622 | - | 1725 | ATA | TAG | -12 |
| | trnH(cac) | tRNA | 6611 | 6676 | - | 66 | | | -1 |
| | nad4 | Coding | 6676 | 8013 | - | 1338 | ATA | TAG | -4 |
| | nad4l | Coding | 8010 | 8303 | - | 294 | ATG | TAA | 1 |
| | trnT(aca) | tRNA | 8305 | 8366 | + | 62 | | | 0 |
| | trnP(cca) | tRNA | 8367 | 8431 | - | 65 | | | -25 |
| | nad6 | Coding | 8407 | 8949 | + | 543 | ATA | TAA | -1 |
| | cob | Coding | 8949 | 10091 | + | 1143 | ATG | TAA | -8 |
| | trnS2(tca) | tRNA | 10084 | 10152 | + | 69 | | | 26 |
| | nad1 | Coding | 10179 | 11140 | - | 962 | ATA | TA | -10 |
| | trnL1(cta) | tRNA | 11131 | 11196 | - | 66 | | | -54 |
| | rrnL | rRNA | 11143 | 12515 | - | 1373 | - | - | -7 |
| | trnV(gta) | tRNA | 12509 | 12573 | - | 65 | | | 0 |
| | rrnS | rRNA | 12574 | 13381 | - | 808 | - | - | 0 |
| | CR Putative | | 13382 | 14185 | | 804 | | | 0 |
| | trnI(atc) | tRNA | 14186 | 14251 | + | 66 | | | 33 |
| | trnQ(caa) | tRNA | 14285 | 14349 | - | 65 | | | -2 |
| | trnM(atg) | tRNA | 14348 | 14414 | + | 67 | | | 27 |
| | nad2 | Coding | 14442 | 15431 | + | 990 | ATA | TAA | -1 |
| | trnW(tga) | tRNA | 15431 | 15494 | + | 64 | | | 29 |
| | trnC(tgc) | tRNA | 15524 | 15592 | - | 69 | | | 0 |
| | trnY(tac) | tRNA | 15593 | 15658 | - | 66 | | | |

Table S3. Best models for each partition used in ML and BI tree searches based on AICc using ModelTest-NG.

| PCGs | Nucleotides | | Amino acids | |
|---|---|---|---|---|
| | RAxML | MrBayes | RAxML | MrBayes |
| *atp6* | GTR+I+G4 | GTR+I+G4 | MTMAM+I+G4m+B | MTMAM+I+G4 |
| *atp8* | GTR+I+G4 | GTR+I+G4 | MTMAM+I+G4m+B | MTMAM+I+G4 |
| *cob* | GTR+I+G4 | GTR+I+G4 | MTMAM+I+G4m+B | MTMAM+I+G4 |
| *cox1* | GTR+I+G4 | GTR+I+G4 | MTZOA+I+G4m+B | MTMAM+I+G4 |
| *cox2* | GTR+I+G4 | GTR+I+G4 | MTZOA+I+G4m+B | MTMAM+I+G4 |
| *cox3* | GTR+I+G4 | GTR+I+G4 | MTZOA+I+G4m+B | MTMAM+I+G4 |
| *nad1* | GTR+I+G4 | GTR+I+G4 | MTZOA+I+G4m+B | MTREV+I+G4 |
| *nad2* | GTR+I+G4 | GTR+I+G4 | MTREV+I+G4m+B | MTREV+I+G4 |
| *nad3* | GTR+I+G4 | GTR+I+G4 | MTMAM+I+G4m+B | MTMAM+G4 |
| *nad4* | GTR+I+G4 | GTR+I+G4 | MTZOA+I+G4m+B | MTREV+I+G4 |
| *nad4l* | GTR+I+G4 | GTR+I+G4 | MTZOA+I+G4m+B | WAG+G4 |
| *nad5* | GTR+I+G4 | GTR+I+G4 | MTZOA+I+G4m+B | VT+I+G4 |
| *nda6* | GTR+I+G4 | GTR+I+G4 | MTREV+I+G4m+B | MTREV+I+G4 |

Table S4. *PAML* results from branch models and branch-site models. For the branch-site models: Class 0: 0 < ω <1, class 1: ω = 1, class 2a: background 0 < ω < 1 & foreground ω > 1; class 2b background ω = 1, foreground ω > 1.

| | Branch models | | | | | | | | Branch-site Models | | | | | | |
|---|---|---|---|---|---|---|---|---|---|---|---|---|---|---|---|
| | Free vs. Fixed | | | Two-ratio vs. Fixed | | | | | Model A vs. Null | | Proportions of sites | | | | |
| PCGs | 2ΔL | p | | 2ΔL | p | | Background w | Foreground w | 2ΔL | p | Class 0 | Class 1 | Class 2a | Class 2b | w |
| *atp6* | 131.299 | <0.001 | * | 2.971 | 0.052 | * | 0.051 | 0.079 | 0 | - | 0.820 | 0.091 | 0.081 | 0.009 | 0.030 |
| *atp8* | 16.532 | 0.041 | * | 0.064 | 1.523 | | 0.165 | 0.188 | 0 | - | 0.720 | 0.178 | 0.082 | 0.020 | 0.097 |
| *cob* | 59.511 | 0.000 | * | 24.649 | <0.001 | * | 0.023 | 0.067 | 0 | - | 0.860 | 0.025 | 0.113 | 0.003 | 0.018 |
| *cox1* | 66.025 | <0.001 | * | 18.385 | <0.001 | * | 0.007 | 0.020 | 0 | - | 0.977 | 0.011 | 0.012 | 0.000 | 0.007 |
| *cox2* | 38.259 | <0.001 | * | 2.116 | 0.095 | | 0.023 | 0.036 | 0.888 | 0.136 | 0.959 | 0.034 | 0.007 | 0.000 | 0.020 |
| *cox3* | 34.404 | <0.001 | * | 16.059 | 0.000 | * | 0.017 | 0.055 | 0 | - | 0.896 | 0.017 | 0.085 | 0.002 | 0.013 |
| *nad1* | 20.845 | 0.015 | * | 6.777 | 0.005 | * | 0.015 | 0.032 | 0 | - | 0.964 | 0.020 | 0.016 | 0.000 | 0.015 |
| *nad2* | 13.169 | 0.071 | | 0.219 | 0.765 | | 0.078 | 0.086 | 0 | - | 0.869 | 0.099 | 0.029 | 0.003 | 0.055 |
| *nad3* | 16.256 | 0.044 | * | 1.711 | 0.130 | | 0.037 | 0.062 | 0 | - | 0.939 | 0.061 | 0.000 | 0.000 | 0.034 |
| *nad4* | 45.587 | <0.001 | * | 2.948 | 0.053 | * | 0.032 | 0.046 | 0 | - | 0.916 | 0.053 | 0.029 | 0.002 | 0.026 |
| *nad4l* | 15.550 | 0.050 | * | 0.055 | 1.655 | | 0.047 | 0.054 | 0 | - | 0.972 | 0.028 | 0.000 | 0.000 | 0.047 |
| *nad5* | 24.782 | 0.005 | * | 2.995 | 0.052 | * | 0.041 | 0.056 | 0 | - | 0.888 | 0.084 | 0.026 | 0.002 | 0.033 |
| *nad6* | 13.830 | 0.065 | | 0.062 | 1.551 | | 0.084 | 0.091 | 0 | - | 0.810 | 0.121 | 0.060 | 0.009 | 0.046 |

Table S5. ANOVA on the effect of sociality (eusocial vs. non-eusocial terminal branches) in predicting dN, dS, and ω, based on results from the free-ratio model of *PAML* of each PCG.

| | dN | | | | dS | | | | | ω | | | | |
|---|---|---|---|---|---|---|---|---|---|---|---|---|---|---|
| PCGs | Slope | F | d.f. | P-value | Slope | F | d.f. | P-value | | Slope | F | d.f. | P-value | |
| atp6 | -0.085 | 0.554 | 1, 6 | 0.485 | -0.224 | 2.175 | 1, 6 | 0.191 | | 0.067 | 0.249 | 1, 6 | 0.635 | |
| atp8 | -0.320 | 0.467 | 1, 6 | 0.520 | 0.213 | 1.113 | 1, 6 | 0.332 | | -0.470 | 0.608 | 1, 6 | 0.471 | |
| cob | 0.231 | 1.444 | 1, 6 | 0.275 | -0.425 | 8.715 | 1, 6 | 0.026 | * | 0.489 | 4.293 | 1, 6 | 0.084 | |
| cox1 | 0.172 | 4.211 | 1, 6 | 0.086 | -0.255 | 4.742 | 1, 6 | 0.072 | | 0.321 | 21.606 | 1, 6 | 0.004 | * |
| cox2 | 0.138 | 0.267 | 1, 6 | 0.624 | 0.056 | 0.064 | 1, 6 | 0.809 | | 0.145 | 0.131 | 1, 6 | 0.730 | |
| cox3 | 0.131 | 1.133 | 1, 6 | 0.328 | -0.328 | 6.521 | 1, 6 | 0.043 | * | 0.374 | 4.120 | 1, 6 | 0.089 | |
| nad1 | 0.042 | 0.444 | 1, 6 | 0.530 | -0.363 | 1.679 | 1, 6 | 0.243 | | 0.223 | 1.318 | 1, 6 | 0.295 | |
| nad2 | -0.035 | 0.181 | 1, 6 | 0.685 | -0.071 | 0.259 | 1, 6 | 0.629 | | -0.002 | 0.000 | 1, 6 | 0.990 | |
| nad3 | 0.506 | 0.567 | 1, 6 | 0.480 | -0.003 | 0.000 | 1, 6 | 0.993 | | 0.883 | 1.216 | 1, 6 | 0.312 | |
| nad4 | 0.035 | 0.292 | 1, 6 | 0.608 | -0.242 | 6.681 | 1, 6 | 0.041 | * | 0.167 | 4.562 | 1, 6 | 0.077 | |
| nad4l | -0.304 | 3.175 | 1, 6 | 0.125 | -0.057 | 0.024 | 1, 6 | 0.884 | | -0.188 | 0.517 | 1, 6 | 0.512 | |
| nad5 | 0.044 | 0.401 | 1, 6 | 0.550 | -0.033 | 0.029 | 1, 6 | 0.870 | | 0.064 | 0.300 | 1, 6 | 0.604 | |
| nad6 | -0.173 | 2.758 | 1, 6 | 0.148 | -0.361 | 0.569 | 1, 6 | 0.479 | | -0.002 | 0.000 | 1, 6 | 0.995 | |

Table S6. Positively selected sites in the mitochondrial PCGs in eusocial *Synalpheus* species. AA: amino acid, SS: secondary structures, RI: reliability index, rSA: relative accessible surface area. * indicates radical replacement of amino acids. *SNP2* results showed that all AA changes were neutral changes. Secondary structures are either a-helix (H), b-sheet (S) or w-loop (L). RI ranges from 0 (low) to 9 (high). rSA are either buried (b, rSA = 0-9%), intermediate (i, rSA = 9-36%), or exposed (e, rSA = 36-100%).

| Sociality | Species group | *Synalpheus sp.* | *cob* 47 | AA | SS (RI) | rSA | *cob* 90 | AA | SS (RI) | rSA |
|---|---|---|---|---|---|---|---|---|---|---|
| Eusocial | *brooksi* | *chacei* | GCT | A | H (7) | b | TTC | F | H (8) | b |
| Non-eusocial | *brooksi* | *carpenteri* | GCA | A | H (7) | b | TTC | F | H (8) | b |
| Eusocial | *paraneptunus* | *microneptunus* | TTC | F * | H (7) | b | GCC | A * | H (8) | b |
| Non-eusocial | *paraneptunus* | *kensleyi* | GCT | A | H (6) | b | TTT | F | H (8) | b |
| Eusocial | *rathbunae* | *filidigitus* | ATC | I | H (6) | b | TTA | L | H (8) | b |
| Eusocial | *rathbunae* | *regalis* | GTA | V | H (7) | b | CTC | L | H (8) | b |
| Non-eusocial | *longicarpus* | *hoetjesi* | GCA | A | H (6) | b | TTC | F | H (8) | b |
| Non-eusocial | *longicarpus* | *pandionis* | GCA | A | H (7) | b | TTT | F | H (8) | b |

| Sociality | Species group | *Synalpheus sp.* | *cob* 214 | AA | SS (RI) | rSA | *cob* 296 | AA | SS (RI) | rSA |
|---|---|---|---|---|---|---|---|---|---|---|
| Eusocial | *brooksi* | *chacei* | ACA | T | L (5) | e | ATT | I | H (8) | b |
| Non-eusocial | *brooksi* | *carpenteri* | AGA | S | L (5) | e | GCA | A | H (8) | b |
| Eusocial | *paraneptunus* | *microneptunus* | AGA | S | L (5) | e | GTT | V | H (8) | b |
| Non-eusocial | *paraneptunus* | *kensleyi* | AGA | S | L (4) | e | GCT | A | H (8) | b |
| Eusocial | *rathbunae* | *filidigitus* | AGT | S | L (5) | e | ATA | M | H (8) | b |
| Eusocial | *rathbunae* | *regalis* | AGA | S | L (5) | e | ATA | M | H (8) | b |
| Non-eusocial | *longicarpus* | *hoetjesi* | AGC | S | L (4) | e | GCC | A | L (4) | b |
| Non-eusocial | *longicarpus* | *pandionis* | AGA | S | L (5) | e | GCA | A | L (4) | b |

| Sociality | Species group | *Synalpheus sp.* | *cox3* 116 | AA | SS (RI) | rSA | *nad1* 163 | AA | SS (RI) | rSA |
|---|---|---|---|---|---|---|---|---|---|---|
| Eusocial | *brooksi* | *chacei* | ATT | I * | L (3) | e | GTG | V * | H (7) | i |
| Non-eusocial | *brooksi* | *carpenteri* | ACC | T | L (4) | e | AGT | S | H (6) | i |
| Eusocial | *paraneptunus* | *microneptunus* | TCT | S | L (4) | e | GGT | G * | H (7) | i |
| Non-eusocial | *paraneptunus* | *kensleyi* | ACT | T | L (4) | e | AGG | S | H (6) | i |
| Eusocial | *rathbunae* | *filidigitus* | AGG | S | L (5) | e | AGA | S | H (6) | i |
| Eusocial | *rathbunae* | *regalis* | AAC | N | L (4) | e | TTG | L * | H (6) | i |
| Non-eusocial | *longicarpus* | *hoetjesi* | ACT | T | L (5) | e | AGA | S | H (6) | e |
| Non-eusocial | *longicarpus* | *pandionis* | ACC | T | L (4) | e | AGC | S | H (6) | i |

| Sociality | *Species group* | *Synalpheus sp.* | *nad2* 184 | AA | SS (RI) | rSA |
|---|---|---|---|---|---|---|
| Eusocial | *brooksi* | *chacei* | ACT | T | H (7) | b |
| Non-eusocial | *brooksi* | *carpenteri* | ACC | T | H (7) | b |

| | | | | | | |
|---|---|---|---|---|---|---|
| Eusocial | *paraneptunus* | *microneptunus* | GCC | A * | H (7) | b |
| Non-eusocial | *paraneptunus* | *kensleyi* | ACC | T | H (7) | b |
| Eusocial | *rathbunae* | *filidigitus* | TCT | S | H (6) | i |
| Eusocial | *rathbunae* | *regalis* | CAA | Q * | H (6) | i |
| Non-eusocial | *longicarpus* | *hoetjesi* | ACC | T | H (7) | b |
| Non-eusocial | *longicarpus* | *pandionis* | ACC | T | H (7) | b |

Table S7. Results from a test of relaxed selection using *RELAX*. "Concat." indicates the concatenated sequences of the 13 PCGs. The alternative model assumes three classes of ω, modified by the selective pressure coefficient *k* between the test branches (eusocial) and reference branches (non-eusocial; "ref"). LR: likelihood ratio.

| | | Against null model | | Alternative model statistics | | | | | | | | | |
|---|---|---|---|---|---|---|---|---|---|---|---|---|---|
| PCG | PCG length (bp) | LR | p | ω 1 test | ω 1 ref | ω 1 % | ω 2 test | ω 2 ref | ω 2 % | ω 3 test | ω 3 ref | ω 3 % | *k* |
| Concat. | | 5.9 | 0.015 | 0 | 0 | 20.569 | 0.041 | 0.026 | 71.013 | 1 | 1 | 8.418 | 0.88 |
| *atp6* | 648 | 0.720 | 0.396 | 0.013 | 0.004 | 83.148 | 0.554 | 0.475 | 16.138 | 233.522 | 970.268 | 0.714 | 0.79 |
| *atp8* | 141 | 1.990 | 0.158 | 0 | 0.001 | 24.976 | 0.190 | 0.324 | 70.858 | 6.255 | 3.479 | 4.167 | 1.47 |
| *cob* | 1131 | 13.600 | 0.000 | 0 | 0 | 84.694 | 0.716 | 0.268 | 15.306 | 1.000 | 1.001 | 0 | 0.25 |
| *cox1* | 1524 | 7.670 | 0.006 | 0.014 | 0.004 | 94.926 | 0.256 | 0.167 | 4.649 | 1.000 | 1.000 | 0.425 | 0.76 |
| *cox2* | 669 | 1.360 | 0.243 | 0.015 | 0.029 | 96.588 | 0.913 | 0.925 | 2.902 | 1.000 | 1.000 | 0.51 | 1.17 |
| *cox3* | 783 | 11.040 | 0.001 | 0 | 0 | 73.888 | 0.275 | 0.093 | 26.112 | 1.001 | 1.001 | 0 | 0.54 |
| *nad1* | 924 | 0.940 | 0.333 | 0.011 | 0.003 | 68.470 | 0.018 | 0.006 | 25.944 | 1.000 | 1.000 | 5.587 | 0.79 |
| *nad2* | 969 | 0.000 | 0.963 | 0 | 0 | 10.362 | 0.056 | 0.054 | 79.424 | 1.000 | 1.000 | 10.215 | 0.99 |
| *nad3* | 339 | 0.500 | 0.479 | 0 | 0 | 80.864 | 0.558 | 0.399 | 19.136 | 1.001 | 1.001 | 0 | 0.64 |
| *nad4* | 1326 | 1.460 | 0.227 | 0.005 | 0 | 84.891 | 0.643 | 0.478 | 15.109 | 1.001 | 1.001 | 0 | 0.60 |
| *nad4l* | 246 | 0.310 | 0.579 | 0.067 | 0.091 | 0 | 0.077 | 0.103 | 100 | 1.114 | 1.101 | 0 | 1.13 |
| *nad5* | 1698 | 2.110 | 0.146 | 0 | 0 | 80.944 | 0.599 | 0.442 | 19.056 | 1.001 | 1.001 | 0 | 0.63 |
| *nad6* | 495 | 0.210 | 0.647 | 0.028 | 0.039 | 23.146 | 0.040 | 0.054 | 66.643 | 1.471 | 1.418 | 10.211 | 1.10 |
| *mean* | | | | | | *62.069* | | | *35.483* | | | *2.448* | |
| *sd* | | | | | | *34.224* | | | *31.938* | | | *3.875* | |
