# Supplementary material for: Eusociality Shapes Convergent Patterns of Molecular Evolution across Mitochondrial Genomes of Snapping Shrimps
Source: Mol Biol Evol. 2020 Nov 19;38(4):1372–83. doi: 10.1093/molbev/msaa297 (PMC8480187; doi:10.1093/molbev/msaa297)
Supplement: msaa297_Supplementary_Data [file msaa297_Supplementary_Data.zip › MBE Comments_20201027_PB-Nov3.docx]

**Response to reviewers**

**MS: MBE-20-0769**

**Title: Eusociality shapes convergent patterns of molecular evolution across mitochondrial genomes of sponge-dwelling snapping shrimps**

**Authors: Chak et al.**

**Response to the editor**

**---------------------------------------------------------------------------------------------------------**

Editors’ comments to the author:

The comments and recommendations from one expert reviewer and associate editor, who acted as a reviewer because another reviewer did not return the review for a long time, are now available for your manuscript. These reviewers judged the reported discoveries to be of high significance, and the potential scientific impact of your work to be medium. They found that the manuscript needs some improvement in text. Editors generally agree with their concerns and recommendations, which led to a designation of high priority.

The recommendation is given assuming that you will be able to satisfactorily address all the reviewers' comments (including my own, as given below). Without a satisfactory response, the priority may decline in the next round of review/consideration.

**Thank you for sending our manuscript for review and taking extra time to review our manuscript. The editor and the reviewer’s comments were very helpful in improving our submission. We agree with the proposed revisions and in the accompanying draft we addressed all of the concerns and recommendations. Please find below a point-by-point response to comments.**

AE’s comments as a reviewer

In this paper, the authors examined the effects of eusociality on the molecular evolution of mitochondrial DNA, using closely related shrimp species with and without eusociality. I think that the results obtained may be rather confirmative but is clearer than the previous studies for the comparisons of distantly related species. My comments are as follows.

Major comments

In this work, it is clear that there is virtually no difference in dN between eusocial species and non-eusocial species, whereas dS is lower in eusocial species than non-eusocial species. Indeed, the dN/dS ratio is commonly used as a proxy of the strength of natural selection. However, in this case, the constancy of dS is intrinsically assumed. Therefore, in this study, the higher dN/dS ratio in the eusocial species does not necessarily mean the relaxed functional importance of mitochondrial proteins but a mere decline in dS. In this context, I can't entirely agree with the reduced effective population sizes in eusocial species (21-33, page 10). If it is so, dN should be higher in eusocial species, although there is a slight such tendency.

**Thank you for the thought-provoking comments, we have modified the lines of reasoning in the discussion, especially the parts related to effective population size and dN/dS. We agree that we need to clarify our rationale, which in part hinges on our result of dN remaining the same or slightly higher, even as dS is lower in eusocial species. However, as the comment points out, the reduced effective population size explanation is not a definitive result and so we have also added qualifiers throughout the text to note the more speculative aspects of our interpretations.**

**As noted in the comment, the dN/dS ratio can be used as a proxy of the strength of natural selection or diversifying (positive) selection, as well as purifying (negative) selection. We agree that when comparing dN/dS ratios, one is comparing the relative levels of dN at the same levels dS. However, dS between the species being compared is not necessarily constant. Instead, the reason that a ratio of dN/dS is used is that one cannot assume the dS to be constant across species.**

**As pointed out, we found a clear decline in dS in eusocial lineages. This is perhaps best explained by the longer generation time in eusocial species. However, dN and dS may be expected to be positively correlated because they are both related to the nucleotide mutation rate (Stoletzki & Eyre-Walker, 2011). Therefore, if the longer generation time in eusocial species is the primary driver of differences in molecular evolution we recovered, we might expect both dS and dN to be lower. Yet, as you have pointed out dN remains constant if not slightly increased in eusocial lineages. This suggests that factors other than generation time may be affecting molecular evolution, and this factor affects dN differently than dS. We do see that we did not explain our thinking clearly and have now clarified this in lines 268-267.**

**What factors can have such an effect? We proposed that it could be the reduced effective population size in eusocial lineages. As we note in the discussion, “lower Ne is associated with a higher probability of fixation of fixation of slightly deleterious mutations, leading to higher nonsynonymous substitution rates” (lines 295-296). Since the effect of lower Ne on dN is in the opposite direction to that of longer generation time (with respect to decreased nucleotide mutation rate), it provides a potential explanation for our observations that dN remained the same between eusocial and non-eusocial lineages, despite the shifts in dS. We have clarified this in lines 299-302: “Curiously, while the reduced Ne in eusocial species would be a plausible explanation for increased dN, the longer generation time in eusocial species may be responsible for decreases in dN. The combined effect of these two factors could explain the lack of a significant trend in dN between eusocial and non-eusocial lineages.” And in lines 305-306: “We suggest that the relatively constant dN and a reduction in dS in eusocial species, as driven by their longer generation time and lower effective populations size, led to an increase in dN/dS ratio (or ω).”**

**Stoletzki, N., & Eyre-Walker, A. (2011). The Positive Correlation between dN/dS and dS in Mammals Is Due to Runs of Adjacent Substitutions. Molecular Biology and Evolution 28(4), 1371-1380. doi:10.1093/molbev/msq320**

Also, I disagree about a higher metabolic rate in eusocial species (54-56, page 10). If it is so, dS should be higher in eusocial species, as a higher metabolic rate results in a higher mutation rate in mitochondrial DNA.

**We agree that higher metabolic rate should result in higher mutation rate in mtDNA. We now clarify this in lines 328-331: “However, higher metabolic rates are expected to increase mutation rate in mtDNA and hence increase dS (Baer, et al. 2007). Our finding of reduced dS suggests that eusocial species may not have higher metabolic rates, at least not as observed from this comparative molecular perspective.”**

**However, oxidative stress in eusocial species that have long lifespan could still lead to positive selection in mitochondrial protein coding genes. We have rearranged this paragraph to clarify the two ideas.**

Minor comments

On 16-19 in page 6, I think that longer PCGS had HIGHER omega, as the decline in k with PCG length (Fig. 4) indicates that PCG length relaxes selection strength.

**We have made this change, it now says “longer PCGs had higher dN, lower dS, and higher ω than shorted PCGs” (line 145).**

The word “the effect of social groups” is ambiguous. Does it mean “the effect of relaxing purifying selection”?

**We have clarified the sentence as “This indicates that the effects of social groups on dN, dS and ω, is more prominent in longer PCGs.” (line 146-147).**

On 54-56 in page 6, the sentence “Pairwise estimations of dN, dS, and omega assume that substitution rates remain constant throughout the phylogenetic history between two species” is not correct. Pairwise estimations of any distances do not assume anything about the rate.

**We have clarified this sentence as “While the above analyses tested the effect of social groups based on pairs of species, the effect of eusociality on dN, dS, and ω can also be tested at branches leading to eusocial species because they may have experienced dramatic changes in life history and selective pressures (Bromham and Leys 2005; Romiguier, et al. 2014).” (lines 169-172).**

On 56 in page 8, I think that “lower omega” should be “higher omega.”

**Change made. Text now says “longer genes exhibited higher ω” (line 238).**

On 18-20 in page 12, I think that all codons are linked to all loci in mtDNA without recombination.

**We have removed this statement, but made our prediction with Hill-Robertson interference more clearly in this paragraph (lines 361-366)**

On 21-23 in page 14, “This analysis does not make any a priori assumptions about rate differences across branches” does not imply that rates are uniform. Note that the uniform distribution is also an “a priori assumption.”

**Thank you for pointing this out, we have removed this statement.**

Reviewers' comments:

Reviewer: 1

Comments to the Author

Review of Manuscript ID MBE-20-0769 “Eusociality shapes convergent patterns of molecular evolution across mitochondrial genomes of sponge-dwelling snapping shrimps”.

The authors investigate patterns of molecular evolution of mitochondrial proteins in eusocial and non-eusocial shrimp in order to determine if eusociality affects rates gene evolution. They find evidence that eusociality may impact rates of molecular evolution.

The study of molecular evolution in social species is interesting. Thus I viewed the subject of the study as quite interesting. The approach and results are also interesting in that they seem to show some consistent patterns of molecular evolution across eusocial species. And I also found the manuscript to be generally well written.

My major concern with this paper is that it only uses mtDNA data, as opposed to whole genome data, for analysis. Whole genome data would be better but are, of course, harder to obtain. The molecular tools available for analyzing non-model species, such as these shrimp, are not as advanced so I do have sympathy that whole genome data are not available for all these species. So, despite this concern, I viewed the research favorably overall, and only have a few minor suggestions.

**We thank the reviewer for their kind comments and for taking the time to evaluate our submission. With respect to whole genome data, this would indeed be preferable, but *Synalpheus* genome sizes are quite large, reaching up to 20gb, which would make a comparative approach computationally and cost prohibitive at this time. We note specific changes and responses to reviewer feedback below.**

Minor Comments

4-29: I think the authors should be more explicit about their predictions here. This will help the reader know where the paper is going and understand the results better. It has been predicted that eusocial species should show lower effective population sizes relative to non-eusocial species. And this would, presumably, lead to selection acting more weakly, and drift acting more strongly, in eusocial species and thus lead to faster rates of molecular evolution. The authors could say this explicitly in the introduction.

**We intended to introduce the matter in an agnostic approach but agree that more structure will assist readers. The broad hypothesis we are testing is the presence of convergent patterns of selection and substitution rates across independent origins of eusociality in shrimps. We have added the following sentence to the final paragraph of the introduction but have reserved the more mechanistic details of these predictions for the discussion where they may be expanded on in greater depth: “Here specifically, we predict that lower effective population size, longer generation times, and shifts in metabolic activity shape rates and patterns of molecular evolution in social shrimps” (lines 85-86).**

Fig 1, 6-8: These ‘regression’ analyses are a little hard for me to understand. First off, I don’t really know if it makes sense to treat the x-axis as a ‘discrete gradient’. Is NN really directly related to NE in the same way that it is related to EE? This seems odd. Perhaps a straight ANOVA among these groups may be better rather than trying to pretend that there is some real clear gradient here.

**We used discrete gradients instead of ANOVA because there is only one species pair of NN, two pairs of NE and one pair of EE. We expand on this approach in lines 461-463, “In treating social groups as an ordinal factor, we expected eusocial-specific substitution rates to have no effect in the NN group, some effects in the NE group, and a strong effect in the EE group.” While the branch length between the species pairs are not the same, we have controlled for the effect of genetic distance. Therefore, we are confident that NN, NE and EE can be treated as a gradient with real biological meaning and that this approach is preferable given sample size limitations.**

I’m also not completely sure what is being compared here. Is this some kind of paired t test where each gene is an controlled unit of comparison across species? Or are the authors ‘controlling’ for genes at all? Or are they just treating all 13 genes in each species as independent units of comparison (which I’m not sure would be right)? The authors do provide some explanation of this analysis. But perhaps some more clarification and justification of this approach would be helpful.

**We controlled for PCG length instead of treating each PCG as independent units. As explained in lines 463-464, “In addition to the factor social groups, our linear model also controlled for the difference in length of PCGs and the effect of genetic distance.” To clarify this for figure 1, we have revised the caption to read: “Pairwise ω, dN, and dS of all 13 mitochondrial PCGs across four sister pairs of *Synalpheus* species of different social groups (NN, NE, EE; where N = non-eusocial and E = eusocial). Lines represent results of linear regressions, which controlled for PCG length and non-independence of loci through nucleotide-based genetic distances…”**

One concern I had was how the linkage of genes within mtDNA would contribute to the observed patterns and potentially bias the results in some way. That is, selection does not operate independently on genes in the mtDNA because selection at one site affects all sites because all genes are linked. How would this be expected to affect the results of this study? How would this differ if whole genomes were used? Some explanation on this issue would be appreciated.

**We thank the reviewer for raising this point and now discuss Hill-Robertson Interference and linkage more clearly in lines 361-374. We also compared mitochondrial and nuclear patterns in lines 258-262: “The convergent patterns in molecular evolution we report here are more likely the consequences of eusociality rather than drivers of complex social behavior. Mitochondrial protein coding genes code for the same conserved cell respiratory functions across all eukaryotes regardless of their ecology and social organizations (Lang, et al. 1999). In contrast to mitochondrial genes, nuclear genes can code for both conserved functions and novel functions associated with the evolution of eusociality.”**

Full email below:

The in-depth review of your manuscript by the editors and the peer reviewers is now complete. Based on their assessment, it is clear that your manuscript requires a substantial revision before it can be considered further for publication in MBE. Comments from the editors and external reviewers are included below.

We invite you to revise your manuscript within 60 days and submit it for further consideration. A delayed submission will be treated as a new submission. If you need an extension, please contact Tom Whitehead by e-mail (EAssist.MBE@gmail.com) before the deadline.

Please note that MBE will be fully Open Access beginning January 1, 2021. All articles accepted for publication from 1 August 2020 will be published Open Access, subject to payment of an Article Processing Charge (APC). Find out more on our Open Access page here: <https://academic.oup.com/mbe/pages/Open_Access>

Most importantly, the revised manuscript will be subject to editorial and external reviews, and its eventual acceptance depends on the reviewers’ and editors' enthusiasm. Note that manuscripts invited to be revised are accepted at a very high rate. But, manuscripts deemed to require more than one major revision are often rejected. So, you must revise it to satisfy all editorial and reviewer concerns. A re-review by original and new reviewers may raise additional concerns, so anticipate them in advance and change thoroughly.

You can create a revision by using the URL: *** PLEASE NOTE: This is a two-step process. After clicking on the link, you will be directed to a webpage to confirm. ***

*** PLEASE NOTE: This is a two-step process. After clicking on the link, you will be directed to a webpage to confirm. ***

<https://mc.manuscriptcentral.com/mbe?URL_MASK=07c716dfab224545aeefac919af0ebb2>

Following is a checklist of needed actions and files.

[#1] Number and respond to each of the comments of the reviewers and the Editor. You must enter these comments in the Authors' Response section on the website when you submit the revision.

[#2] Please follow the Author Guidelines at: (<https://academic.oup.com/mbe/pages/General_Instructions>

In the File Upload section of the submission website, please provide the following:

[#3] Upload a file in DOC/DOCX format, RTF format, or LaTeX format containing the text, tables and figure legends. If you are submitting a LaTex/Tex file, please include accompanying .tex files (e.g., .bib, .sty, .cls,.bst).

[#4] Upload files containing high-resolution figures – this does not apply to any supplementary figures. If you have color figures, please confirm which ones are to be published in color in print. If you have color figures that are to be published color online only, please ensure the figure legends do not refer to color. Images are required as high-resolution files (600-1200 dpi for line drawings and 350 dpi for color and half-tone artwork). Most formats are acceptable, including eps, tiff, pdf, jpg, and Powerpoint.

[#5] Upload a copy of the manuscript in PDF (for “Advance Access”) that contains the complete manuscript, including text, any tables, figure legends, and figures, but excluding any supplementary material.

[#6] Upload any supplementary information files. There is no limit on supplementary files. All supplementary information, including supplementary tables and supplementary figure legends, must be uploaded separately as "supplementary files" and not placed in the manuscript. (<https://academic.oup.com/mbe/pages/Supplementary_Information>).

[#7] Double-check the format of citations and references to ensure that they meet MBE requirements (<https://academic.oup.com/mbe/pages/General_Author_Guidelines#References>). Failure to do so will result in a delay in manuscript processing.

[#8] Double-check the format for revisions. Articles, Letters, Brief Communications, Perspectives, Reviews, and Protocols all have different format requirements.

Articles must have headings in order: Introduction, Results, Discussion, and Materials and Methods. Results and Discussion may be combined. Subsections are allowed throughout.

Brief Communications must be in the resources category.

(<https://academic.oup.com/mbe/pages/manuscript_types>)

[#9] To ensure brevity, please make use of unlimited supplementary files to keep manuscripts below 20 pages using 1.5 line spacing for the main text (excluding references, figure legends, or tables). If the manuscript is longer, please justify the extent in response to review.

[#10] Your original files will be available to you when you upload your revised manuscript. Please delete any redundant files before completing the submission.

We look forward to receiving a revised version of the manuscript for further consideration.

Sincerely,

Board of Editors

Molecular Biology and Evolution

Editors’ comments to the author:

The comments and recommendations from one expert reviewer and associate editor, who acted as a reviewer because another reviewer did not return the review for a long time, are now available for your manuscript. These reviewers judged the reported discoveries to be of high significance, and the potential scientific impact of your work to be medium. They found that the manuscript needs some improvement in text. Editors generally agree with their concerns and recommendations, which led to a designation of high priority.

The recommendation is given assuming that you will be able to satisfactorily address all the reviewers' comments (including my own, as given below). Without a satisfactory response, the priority may decline in the next round of review/consideration.

AE’s comments as a reviewer

In this paper, the authors examined the effects of eusociality on the molecular evolution of mitochondrial DNA, using closely related shrimp species with and without eusociality. I think that the results obtained may be rather confirmative but is clearer than the previous studies for the comparisons of distantly related species. My comments are as follows.

Major comments

In this work, it is clear that there is virtually no difference in dN between eusocial species and non-eusocial species, whereas dS is lower in eusocial species than non-eusocial species. Indeed, the dN/dS ratio is commonly used as a proxy of the strength of natural selection. However, in this case, the constancy of dS is intrinsically assumed. Therefore, in this study, the higher dN/dS ratio in the eusocial species does not necessarily mean the relaxed functional importance of mitochondrial proteins but a mere decline in dS. In this context, I can't entirely agree with the reduced effective population sizes in eusocial species (21-33, page 10). If it is so, dN should be higher in eusocial species, although there is a slight such tendency. Also, I disagree about a higher metabolic rate in eusocial species (54-56, page 10). If it is so, dS should be higher in eusocial species, as a higher metabolic rate results in a higher mutation rate in mitochondrial DNA.

Minor comments

On 16-19 in page 6, I think that longer PCGS had HIGHER omega, as the decline in k with PCG length (Fig. 4) indicates that PCG length relaxes selection strength. The word “the effect of social groups” is ambiguous. Does it mean “the effect of relaxing purifying selection”?

On 54-56 in page 6, the sentence “Pairwise estimations of dN, dS, and omega assume that substitution rates remain constant throughout the phylogenetic history between two species” is not correct. Pairwise estimations of any distances do not assume anything about the rate.

On 56 in page 8, I think that “lower omega” should be “higher omega.”

On 18-20 in page 12, I think that all codons are linked to all loci in mtDNA without recombination.

On 21-23 in page 14, “This analysis does not make any a priori assumptions about rate differences across branches” does not imply that rates are uniform. Note that the uniform distribution is also an “a priori assumption.”

Reviewers' comments:

Reviewer: 1

Comments to the Author

Review of Manuscript ID MBE-20-0769 “Eusociality shapes convergent patterns of molecular evolution across mitochondrial genomes of sponge-dwelling snapping shrimps”.

The authors investigate patterns of molecular evolution of mitochondrial proteins in eusocial and non-eusocial shrimp in order to determine if eusociality affects rates gene evolution. They find evidence that eusociality may impact rates of molecular evolution.

The study of molecular evolution in social species is interesting. Thus I viewed the subject of the study as quite interesting. The approach and results are also interesting in that they seem to show some consistent patterns of molecular evolution across eusocial species. And I also found the manuscript to be generally well written.

My major concern with this paper is that it only uses mtDNA data, as opposed to whole genome data, for analysis. Whole genome data would be better but are, of course, harder to obtain. The molecular tools available for analyzing non-model species, such as these shrimp, are not as advanced so I do have sympathy that whole genome data are not available for all these species. So, despite this concern, I viewed the research favorably overall, and only have a few minor suggestions.

Minor Comments

4-29: I think the authors should be more explicit about their predictions here. This will help the reader know where the paper is going and understand the results better. It has been predicted that eusocial species should show lower effective population sizes relative to non-eusocial species. And this would, presumably, lead to selection acting more weakly, and drift acting more strongly, in eusocial species and thus lead to faster rates of molecular evolution. The authors could say this explicitly in the introduction.

Fig 1, 6-8: These ‘regression’ analyses are a little hard for me to understand. First off, I don’t really know if it makes sense to treat the x-axis as a ‘discrete gradient’. Is NN really directly related to NE in the same way that it is related to EE? This seems odd. Perhaps a straight ANOVA among these groups may be better rather than trying to pretend that there is some real clear gradient here. I’m also not completely sure what is being compared here. Is this some kind of paired t test where each gene is an controlled unit of comparison across species? Or are the authors ‘controlling’ for genes at all? Or are they just treating all 13 genes in each species as independent units of comparison (which I’m not sure would be right)? The authors do provide some explanation of this anlaysis. But perhaps some more clarification and justification of this approach would be helpful.

One concern I had was how the linkage of genes within mtDNA would contribute to the observed patterns and potentially bias the results in some way. That is, selection does not operate independently on genes in the mtDNA because selection at one site affects all sites because all genes are linked. How would this be expected to affect the results of this study? How would this differ if whole genomes were used? Some explanation on this issue would be appreciated.
